# PWL1, a G‐type lectin receptor‐like kinase, positively regulates leaf senescence and heat tolerance but negatively regulates resistance to *Xanthomonas oryzae* in rice

**DOI:** 10.1111/pbi.14150

**Published:** 2023-08-14

**Authors:** Jiangmin Xu, Chunlian Wang, Fujun Wang, Yapei Liu, Man Li, Hongjie Wang, Yuhan Zheng, Kaijun Zhao, Zhiyuan Ji

**Affiliations:** ^1^ National Key Facility for Crop Gene Resources and Genetic Improvement Institute of Crop Sciences, Chinese Academy of Agricultural Sciences Beijing China; ^2^ Institute of Rice Research, Guangdong Academy of Agricultural Sciences Guangzhou China

**Keywords:** lectin receptor‐like kinase, rice (*Oryza sativa* L.), PWL1, leaf senescence, reactive oxygen species, chloroplast development

## Abstract

Plant leaf senescence, caused by multiple internal and environmental factors, has an important impact on agricultural production. The lectin receptor‐like kinase (LecRLK) family members participate in plant development and responses to biotic and abiotic stresses, but their roles in regulating leaf senescence remain elusive. Here, we identify and characterize a rice *premature withered leaf 1* (*pwl1*) mutant, which exhibits premature leaf senescence throughout the plant life cycle. The *pwl1* mutant displayed withered and whitish leaf tips, decreased chlorophyll content, and accelerated chloroplast degradation. Map‐based cloning revealed an amino acid substitution (Gly412Arg) in LOC_Os03g62180 (*PWL1*) was responsible for the phenotypes of *pwl1*. The expression of *PWL1* was detected in all tissues, but predominantly in tillering and mature leaves. *PWL1* encodes a G‐type LecRLK with active kinase and autophosphorylation activities. PWL1 is localized to the plasma membrane and can self‐associate, mainly mediated by the plasminogen‐apple‐nematode (PAN) domain. Substitution of the PAN domain significantly diminished the self‐interaction of PWL1. Moreover, the *pwl1* mutant showed enhanced reactive oxygen species (ROS) accumulation, cell death, and severe DNA fragmentation. RNA sequencing analysis revealed that *PWL1* was involved in the regulation of multiple biological processes, like carbon metabolism, ribosome, and peroxisome pathways. Meanwhile, interfering of biological processes induced by the *PWL1* mutation also enhanced heat sensitivity and resistance to bacterial blight and bacterial leaf streak with excessive accumulation of ROS and impaired chloroplast development in rice. Natural variation analysis indicated more variations in *indica* varieties, and the vast majority of *japonica* varieties harbour the *PWL1* ^*Hap1*^ allele. Together, our results suggest that PWL1, a member of LecRLKs, exerts multiple roles in regulating plant growth and development, heat‐tolerance, and resistance to bacterial pathogens.

## Background

Leaf senescence is the final stage of plant leaf development, accompanied by the degradation of intracellular organelles and the decomposition of macromolecules to relocate nutrients from senescing leaves into developing tissues and/or storage organs (Lim *et al*., 2007). During leaf senescence, leaf cells undergo a series of dramatic changes in cellular and physiological processes, including the degradation of chlorophylls, nucleic acids, lipids, proteins, and other cellular components, reactive oxygen species (ROS) accumulation, and subsequently programmed cell death (PCD) and changes in leaf colour (Lim *et al*., 2007; Woo *et al*., 2019). Early leaf senescence (ELS) makes plants senesce earlier, which can occur at the seedling stage (Leng *et al*., 2017), tillering stage (Liang *et al*., 2014; Xu *et al*., 2022b) or heading stage (Ke *et al*., 2019), and shortens the vegetative growth period, resulting in reduced grain yield and quality (Yang *et al*., 2016). Conversely, delaying leaf senescence (DLS) enables plants to retain greenness during the senescence phase, which can prolong photosynthetic activity, increase biomass accumulation, and sometimes improve grain filling and crop yield (Rivero *et al*., 2007). Thus, understanding the leaf senescence mechanisms through the isolation and characterization of ELS and DLS mutants is of great significance for crop breeding.

Rice (*Oryza sativa* L.) is one of the most important crops worldwide and is consumed by nearly half of the global population. To date, more than 227 senescence‐associated genes (SAGs) have been identified and cloned in rice according to the LSD 4.0 database (LSD, https://ngdc.cncb.ac.cn/lsd/) (Cao *et al*., 2022; Woo *et al*., 2019). These genes play roles in various biological processes, such as chloroplast development, chlorophyll degradation, hormone signal transduction, and protease transport metabolism (Xu *et al*., 2022a; Zhang *et al*., 2021). For example, *NYC1*, *NYC3*, and *PGL* are involved in chloroplast development. *NYC1* encodes a short‐chain dehydrogenase/reductase located in the chloroplast, which regulates leaf senescence by participating in the degradation of the photosystem II light harvesting complex and grana (Kusaba *et al*., 2007). *NYC3* encodes an α/β folding hydrolase family protein; mutation of *NYC3* causes a stay‐green phenotype during leaf senescence (Morita *et al*., 2009). *PGL* encodes chlorophyllide a oxygenase 1 required for chlorophyll biosynthesis, which regulates leaf senescence and indirectly affects rice grain yield and quality (Yang *et al*., 2016). *RLS1* encodes a NB (nucleotide‐binding site)‐containing protein with an ARM (armadillo) domain at the carboxyl terminus, which regulates leaf senescence by affecting the degradation process of chlorophyll (Jiao *et al*., 2012). It was reported that the *OsNAP*, *OsFBK12*, and *OsDOS* genes are involved in the phytohormone pathway in rice leaf senescence. *OsNAP* encodes a plant‐specific NAC transcription activator, which is an important member of the abscisic acid (ABA)‐mediated senescence signalling pathway and specifically induced by ABA (Liang *et al*., 2014). *OsFBK12* encodes an F‐box protein with a kelch repeat motif, which regulates leaf senescence via the ethylene (ETH) signalling pathways (Chen *et al*., 2013b). *OsDOS* encodes a CCCH‐type zinc finger protein, which regulates leaf senescence by negatively regulating the jasmonic acid (JA) signalling pathway (Kong *et al*., 2006). The SAGs that play roles in energy metabolism pathways include *LST1* and *PLS3*. *LST1* encodes a nicotinate phosphoribosyltransferase (OsNaPRT1), regulating leaf senescence by affecting the content of nicotinamide (Wu *et al*., 2016). *PLS3* encodes an O‐methyltransferase in the melatonin biosynthetic pathway; disruption of *PLS3* can trigger leaf senescence as a result of decreased melatonin production (Hong *et al*., 2018). Moreover, some other SAGs have been identified, such as *ES1* (Rao *et al*., 2015), *OsSWEET5* (Zhou *et al*., 2014), *OsNAC2* (Mao *et al*., 2017), *DELA* (Leng *et al*., 2017), YPD1 (Chen *et al*., 2021), *ONAC054* (Sakuraba *et al*., 2020), *ESL1* (Xu *et al*., 2022b). However, the association between plant leaf senescence and lectin receptor‐like kinases (LecRLKs) remains elusive.

The LecRLKs constitute a subfamily of receptor‐like kinases (RLKs) characterized by an extracellular lectin domain. A LecRLK contains three structural domains: an extracellular lectin domain, a transmembrane (TM) domain and an intracellular kinase domain (Sun *et al*., 2020). There are 75 LecRLKs in *Arabidopsis* and 173 members in rice (Vaid *et al*., 2012). Based on the diversity of lectin domains, LecRLKs are classified into three groups: L‐type, G‐type, and C‐type (Sun *et al*., 2020). The L‐type LecRLKs are characterized by a legume‐lectin domain that possesses a typical β‐sandwich fold structure and has a conserved hydrophobic cavity for binding to hydrophobic ligands (Bellande *et al*., 2017; Sun *et al*., 2020). A G‐type LecRLK contains an α‐mannose binding bulb‐lectin domain (B‐lectin), an S‐locus glycoprotein (SLG) domain, a plasminogen‐apple‐nematode (PAN) domain and/or an epidermal growth factor domain extracellularly (Vaid *et al*., 2012). The C‐type LecRLKs contain carbohydrate recognition domains that bind carbohydrate structure in a calcium (Ca^2+^)‐dependent manner and mediate innate immune responses in mammals (Cambi *et al*., 2005), but the plant C‐type LecRLKs are the smallest group, represented by only one member found in both *Arabidopsis* and rice (Sun *et al*., 2020). Investigations have shown that LecRLKs play crucial and versatile roles in responses to pathogens and insect pests (Liu *et al*., 2015; Pi *et al*., 2022; Wang *et al*., 2019), and responses towards various abiotic stresses (Li *et al*., 2014; Sun *et al*., 2013), plant–mycorrhizal interaction (Labbé *et al*., 2019), hormone signalling (Luo *et al*., 2017; Zhang *et al*., 2020), pollen development (He *et al*., 2021b; Peng *et al*., 2020; Wan *et al*., 2008; Zhang *et al*., 2020), seed germination (Cheng *et al*., 2013), seed yield and the source–sink relationship (Xiao *et al*., 2021), fibre development (Zuo *et al*., 2004), and dark‐induced leaf senescence (Chen *et al*., 2013a).

Heat stress is one of the most destructive environmental stresses that seriously affects crop growth and productivity (Janni *et al*., 2020; Lobell *et al*., 2011). When plants are subjected to heat stress, metabolic imbalance occurs, ROS accumulation occurs, and PCD is activated, which can lead to plant death in severe cases (Li *et al*., 2021; Vu *et al*., 2019; Xia *et al*., 2022). However, there are few studies on heat stress‐induced leaf senescence in rice, and the potential molecular mechanisms between leaf senescence and abiotic/biotic stress remains unclear. In this study, we identified a rice recessive mutant exhibiting premature leaf senescence and withering, named *premature withered leaf 1* (*pwl1*). And the *pwl1* mutant also displayed sensitivity to heat stress, but resistance to bacterial pathogens, *Xanthomonas oryzae* pv. *oryzae* (*Xoo*) and *X*. *oryzae* pv. *oryzicola* (*Xoc*). Map‐based cloning results show that the mutant is due to a missense mutation in LOC_Os03g62180 (*PWL1*), which encodes a G‐type LecRLK. Here, we reveal a ubiquitous LecRLK that plays important roles in the regulation of leaf senescence, heat tolerance, and disease resistance in rice.

## Results

### The *pwl1* mutant exhibits premature leaf senescence phenotype, dark‐induced senescence, and defect in chloroplast development

**Figure 1:**
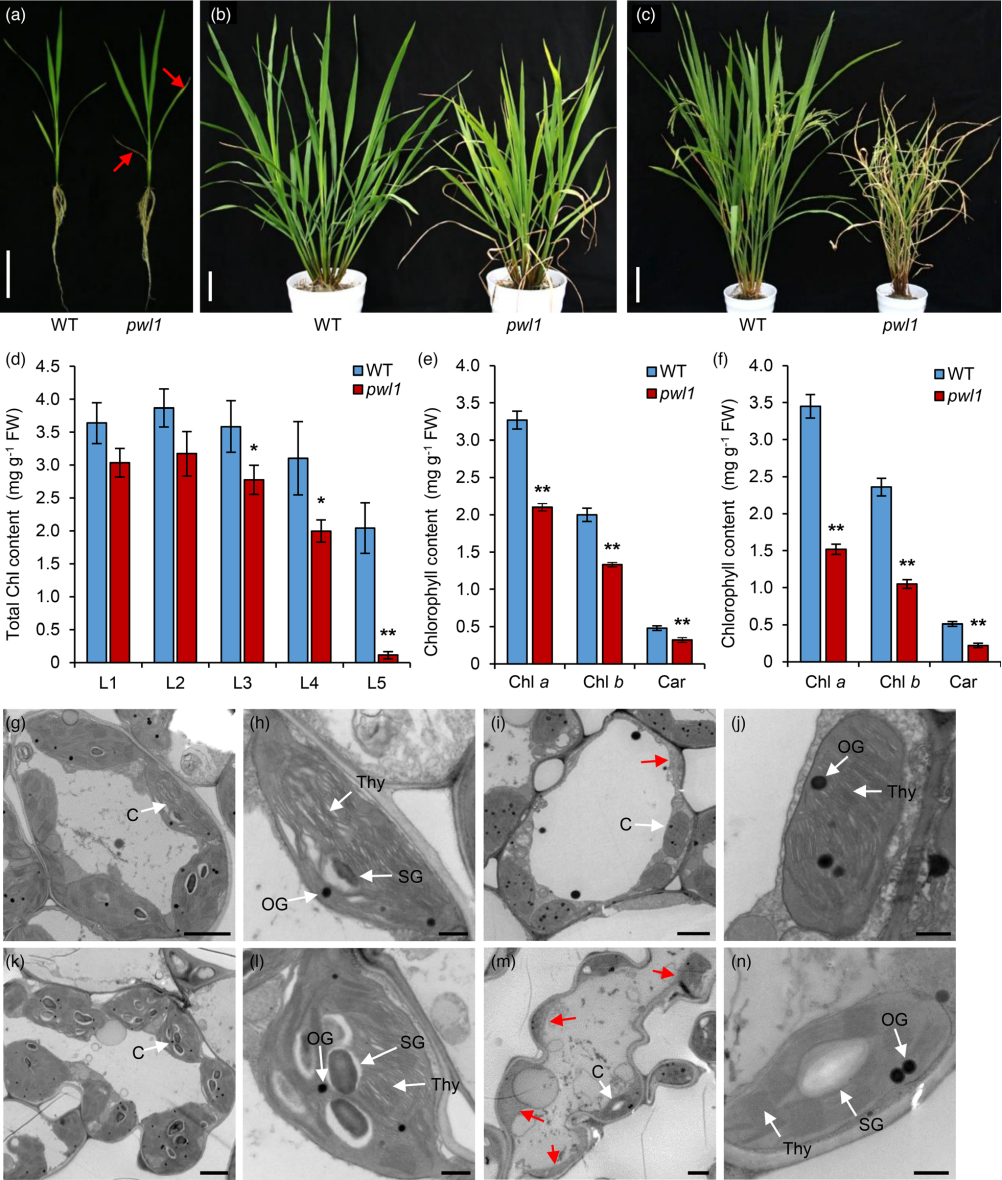
Phenotype characterization and chloroplast ultrastructural analysis of the rice *pwl1* mutant. (a) Phenotypes of wild type (WT) and *pwl1* at the seedling stage. Arrows indicate the withered leaves and whitish leaf tips in *pwl1*. Bar, 8 cm. (b) Phenotypes of WT and *pwl1* at the tillering stage. Bar, 10 cm. (c) Phenotypes of WT and *pwl1* at the heading stage. Bar, 20 cm. (d) Comparison of total chlorophyll content between WT and *pwl1* at the seedling stage. The L1, L2, L3, L4, and L5 represent the first, second, third, fourth, and fifth leaves from the top of the plants, respectively. (e) The chlorophyll content of WT and *pwl1* at the tillering stage. (f) The chlorophyll content of WT and *pwl1* at the heading stage. (g–j) Transmission electron microscopy (TEM) was used to detect the ultrastructural chloroplasts in WT leaves (g, h) and *pwl1* leaves (i, j) at the tillering stage. (k–n) Ultrastructure of chloroplasts in WT leaves (k, l) and *pwl1* leaves (m, n) at the heading stage. C, chloroplast; OG, osmiophilic plastoglobuli; SG, starch granule; Thy, thylakoid. Red arrows indicate degraded chloroplasts. Bars: 2 μm in (g, i, k, m), 0.5 μm in (h, j, l, n). Data are means ± SD (*n* = 3). *P < 0.05; **P < 0.01 (Student's *t*‐test).

Under natural field conditions in Beijing, the lower leaves of *pwl1* exhibited withered leaves and whitish leaf tips approximately 40 days after germination as compared with the wild‐type (WT) plants (Figure 1a). This withered leaf phenotype aggravated along with the growth of plants (Figure 1b), reaching severer leaf withering and premature senescence at the heading stage (Figure 1c). At the heading stage, the plant height of *pwl1* was much shorter (Figure 1c), and the lengths of the internodes I, II, III, IV, and V of *pwl1* were shortened by 32.4%, 16.1%, 63.1%, 40.2%, and 51.4%, respectively (Figure S1a,b). Compared with the WT, the primary and second branch numbers per panicle, the tiller number, seed‐setting rate, and 1000‐grain weight were also reduced in *pwl1* (Table S1). Moreover, the iodine‐potassium iodide (I_2_‐KI) staining assay indicated that the pollen grains of *pwl1* were defective and had low fertility compared with the WT (Figure S1c). We conclude that the premature leaf senescence of *pwl1* has an overall negative effect on plant growth and development.

Darkness is one of the best‐known external stimuli of leaf senescence, and dark stress can significantly promote premature leaf senescence in plants (Liebsch and Keech, 2016). We subsequently examined the phenotypes of the WT and *pwl1* plants under dark stress. For dark‐induction experiments, 2‐week‐old seedlings of WT and *pwl1* were transferred into complete darkness. After 6 day of dark incubation (DDI), most of the *pwl1* mutant plants withered rapidly and then died, with a survival rate of only 10.8%, while only a few of the WT plants withered, with a survival rate of about 92.3% (Figure S2a,b). Meanwhile, the accelerated leaf senescence phenotype of *pwl1* was observed in the detached leaves. After 5 DDI, the leaf discs of *pwl1* turned yellow faster than the WT, which was consistent with the total chlorophyll (Chl) content being significantly lowered than that in the WT (Figure S2c,d). These results suggested that the *pwl1* leaf senescence was more sensitive to dark induction.

Since the *pwl1* mutant produced pale yellow leaves from the tillering stage, we measured the Chl contents in *pwl1* and WT plants at different growth stages. At the seedling stage (five‐leaf stage), the total Chl contents of the third leaf, the fourth leaf, and the fifth leaf of *pwl1* were significantly reduced compared with those in the WT leaves, especially the fifth leaf, which was essentially devoid of chlorophyll (Figure 1d). Moreover, the Chl *a*, Chl *b*, and carotenoid (Car) contents in the *pwl1* leaves were remarkably lower than those of the WT at the tillering and heading stages (Figure 1e,f). We also observed the chloroplast ultrastructure of the WT and *pwl1* leaves using transmission electron microscopy (TEM) at the tillering and heading stages. At the tillering stage, the chloroplasts of WT were normal and contained well‐structured thylakoid grana lamellae (Figure 1g,h). By contrast, in the *pwl1* leaves, the thylakoid grana lamellae were abnormal, the chloroplasts were degraded, and they produced more osmiophilic granules (OG) than those in the WT leaves (Figure 1i,j). At the heading stages, the chloroplasts of WT were filled with starch granules (SG) and dense thylakoid grana (Figure 1k,l). However, in the *pwl1* leaves, thylakoid grana lamellae were loose, the chloroplast structure was abnormal, degraded chloroplasts were clearly visible, and the number of chloroplasts was reduced compared to WT (Figure 1m,n). These results indicated that the lower Chl content and pale‐yellow leaf phenotypes of the *pwl1* mutant were caused by chloroplast degradation, suggesting a vital role for *PWL1* in chloroplast development.

### Map‐based cloning of *PWL1*

**Figure 2:**
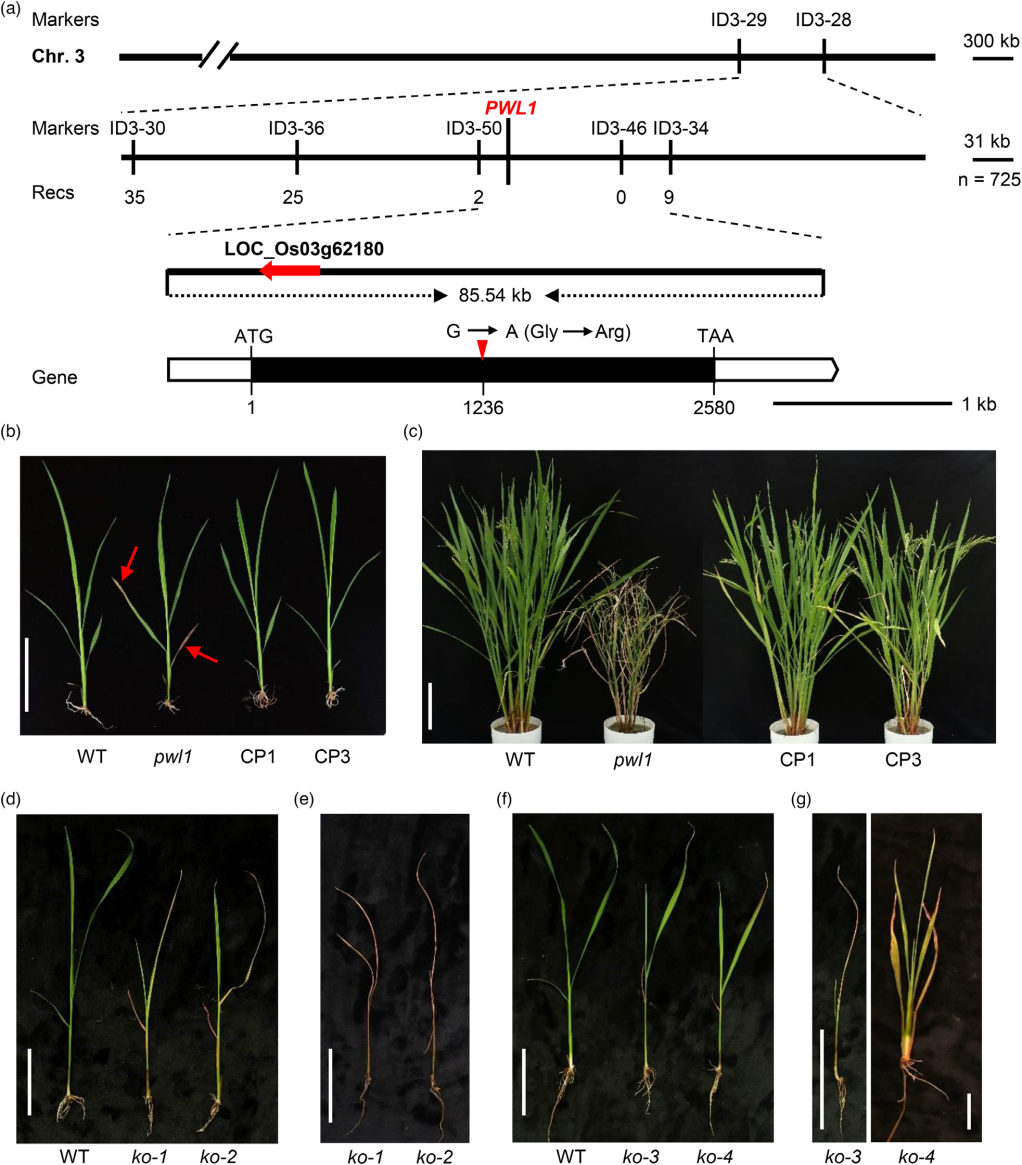
Map‐based cloning and functional characterization of *PWL1*. (a) Map‐based cloning of *PWL1*. *PWL1* was fine‐mapped to an 85.54 kb genomic region between markers ID3‐50 and ID3‐34. The numbers below the primers correspond to the number of recombinants. The white boxes and black box represent untranslated regions and exon, respectively. The red arrow indicates the mutation site. The start codon (ATG) and stop codon (TAA) are also indicated. (b) The plant morphology among wild type (WT), *pwl1*, and complementation transgenic lines (CP1 and CP3) at the seedling stage. Arrows indicate the withered leaves and whitish leaf tips in *pwl1*. Bar, 6 cm. (c) The gross morphologies of WT, *pwl1*, CP1, and CP3. Bar, 18 cm. (d) Phenotypes of WT, *PWL1*‐edited lines (generated by CRISPR/Cas9) *ko‐1* and *ko‐2* plants at the three‐leaf stage. Bar, 6 cm. (e) Phenotypes of *ko‐1* and *ko‐2* plants at the four‐leaf stage. Bar, 6 cm. (f) Phenotypes of WT, *PWL1*‐edited lines *ko‐3* and *ko‐4* plants at the three‐leaf stage. Bar, 6 cm. (g) Phenotypes of *ko‐3* plant at the four‐leaf stage and *ko‐4* plant at 60 days after sowing. Bars, 10 cm.

To reveal the genetic control of the *pwl1* phenotype, we crossed *pwl1* with the *japonica* rice Nipponbare (NIP) and the *indica* cultivar JG30, respectively. All F_1_ plants exhibited the WT phenotype. In the F_2_ segregating populations, the normal and mutant plants displayed a typical Mendelian 3 : 1 segregation ratio (Table S2), indicating that the premature leaf senescence phenotype of *pwl1* was controlled by a single recessive locus. Using 21 mutant individuals derived from the cross of NIP/*pwl1* for genetic linkage analysis, the *PWL1* locus was mapped to chromosome 3 between the InDel markers ID3‐29 and ID3‐28 (Figure 2a). For fine mapping of *PWL1*, five new InDel markers were developed within the target interval using the reference genome sequences from NIP and JG30 (Table S3). Using 725 mutant‐type F_2_ individuals, *PWL1* was finally narrowed down to an 85.54 kb region between the markers ID3‐50 and ID3‐34 (Figure 2a). This region contains 17 annotated open reading frames (ORFs) (Table S4, http://rice.plantbiology.msu.edu/). Sequencing analysis revealed a single nucleotide substitution from G to A within LOC_Os03g62180, resulting in an amino acid substitution from glycine (Gly) to arginine (Arg) in the 412th residue in *pwl1* (Figure 2a).

To determine that the mutation in LOC_Os03g62180 is responsible for the *pwl1* mutant phenotype, we performed a genetic complementation by introducing a 4.9 kb WT genomic DNA fragment containing the entire coding region of LOC_Os03g62180 along with 1.6 kb upstream sequence and 0.7 kb downstream sequence into *pwl1*. Ten transgenic lines were obtained, and all of them fully rescued the premature leaf senescence phenotype of *pwl1*, showing a normal leaf phenotype (Figure S3a and Figure 2b,c). As expected, the transgene positive lines (including CP1 and CP3) were heterozygous for the *pwl1* mutation site in the T0 transgenic (Figure S3b). Additionally, the plant height, tiller number, second branch number per panicle, panicle length, seed‐setting rate, and 1000‐grain weight of CP1 and CP3 were recovered to the WT level (Table S1).

### *PWL1* encodes a G‐type lectin receptor kinase with auto‐phosphorylase activity

**Figure 3:**
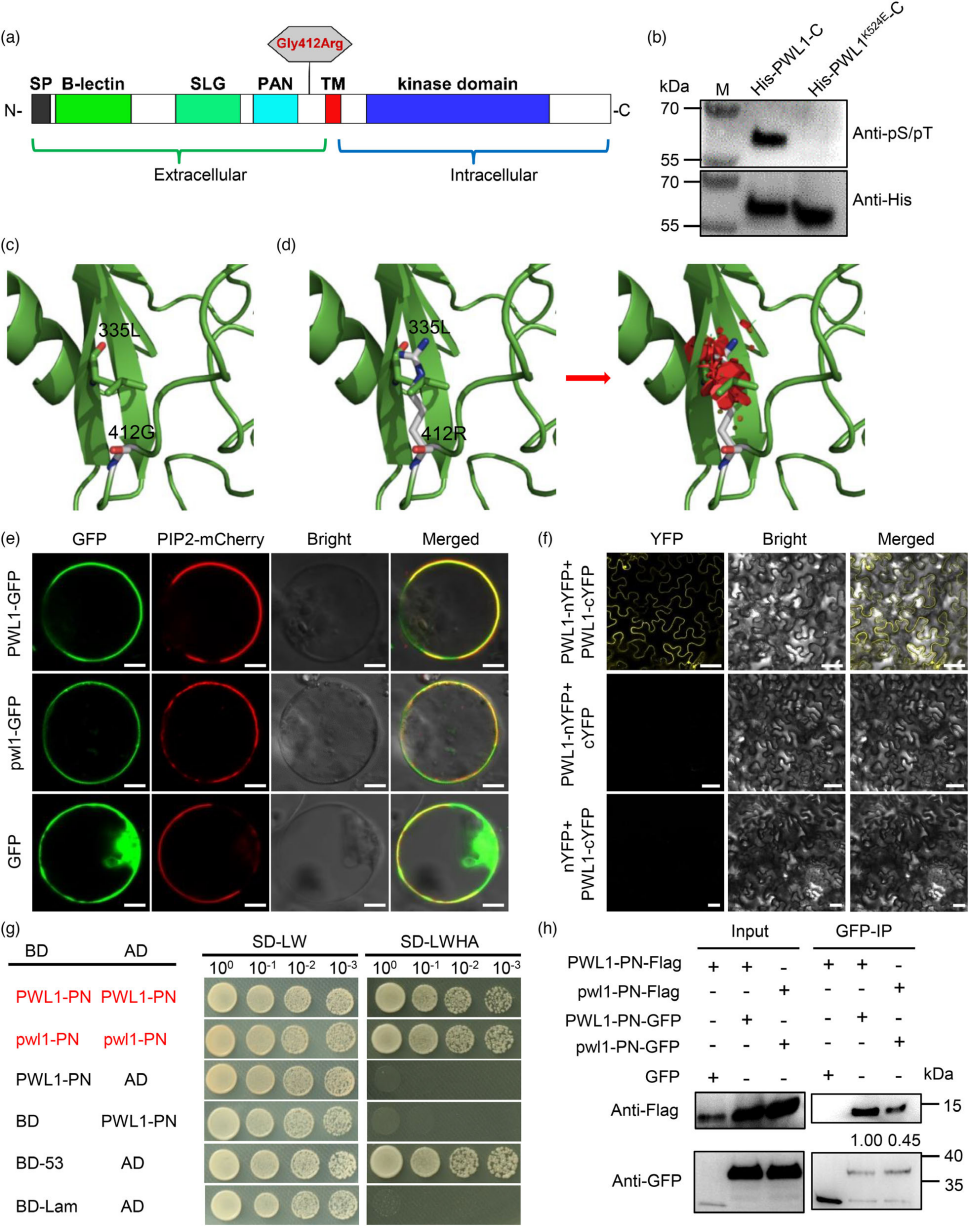
Bioinformatics analysis, kinase activity, and protein properties of the PWL1 protein. (a) Schematic diagram of the PWL1 protein. SP, signal peptide; SLG, S‐locus glycoprotein domain; TM, transmembrane domain. The hexagon indicates the position of the point mutation of PWL1 in the *pwl1* mutant. (b) PWL1 kinase activity. Recombinant polyhistidine (His)‐tagged PWL1‐C and PWL1^K524E^‐C (kinase‐dead variant) proteins were purified from *E. coli* and auto‐phosphorylation was detected using an Anti‐phospho (Ser/Thr) Phe antibody, with Anti‐His being used as a loading control. (c) Partial structure model of the PWL1 protein. L, leucine; G, glycine. (d) Partial structure model of the pwl1 protein (G412R). R, arginine. The arrow indicates that the Gly412Arg substitution induced a steric clash with β‐sheet (332–338) of PAN domain, the red module indicates the section on steric clash. (e) Subcellular localization of PWL1‐GFP and pwl1‐GFP fusion proteins in rice sheath protoplasts. The PIP2‐mCherry fusion protein served as a plasma membrane marker. p35S:GFP was used as the control. Bars, 5 μm. (f) PWL1 interacts with itself in the BiFC assay performed in leaf epidermal cells of *Nicotiana benthamiana*. Bars, 20 μm. (g) PN fragment harbouring the PAN domain (aa 329–434) interacts with itself in the Y2H assay. Yeast cells were grown on synthetically defined (SD) medium lacking leucine and tryptophan (–LW) and SD medium lacking leucine, tryptophan, histidine, and adenine (–LWHA). (h) PN fragment interacts with itself in the Co‐IP assay. Total protein from rice protoplasts transfected with the indicated plasmid combinations was extracted and subjected to immunoprecipitation with anti‐GFP magnetic beads, followed by Western blot analysis using Anti‐GFP or Anti‐Flag antibodies. The relative contents of proteins were measured using Image J software and indicated with numbers below the bands.

Sequence analysis indicated that the coding sequence (CDS) of *PWL1* consists of 2580 nucleotides, putatively encoding an 859‐amino acid protein. According to annotation by the Rice Genome Annotation Project (http://rice.plantbiology.msu.edu/), *PWL1* encodes a lectin protein kinase family protein. SMART (http://smart.embl‐heidelberg.de/) and NCBI‐conserved domain database (CDD) (https://www.ncbi.nlm.nih.gov/Structure/cdd/wrpsb.cgi) analyses showed that PWL1 is a putative G‐type LecRLK consisting of an extracellular signal peptide (SP) domain, a classic B‐lectin domain, an SLG domain, a PAN domain, a TM domain, and an intracellular serine/threonine kinase (STK) domain (Figure 3a). To determine whether PWL1 possesses kinase activity, we purified the recombinant protein His‐PWL1‐C (intracellular domain, amino acids 460–859) and the kinase‐dead mutant variant His‐PWL1^K524E^‐C, which is the conserved ATP‐binding site required for kinase activity, from *Escherichia coli*, as we were unable to obtain the full length PWL1 protein. An *in vitro* kinase assay showed that phosphorylation signals were observed for PWL1‐C but not for PWL1^K524E^‐C (Figure 3b and Figure S4a,b), supporting that PWL1 is an active kinase and can be auto‐phosphorylated.

In the *pwl1* mutant, the Gly412Arg substitution occurred in the extracellular region, closer to the PAN domain. Prediction of protein structures showed that the longer side chains of Arg induced steric clashes with β‐sheet (332–338) of the PAN domain, which could severely disrupt PWL1 function (Figure 3c,d). To further verify the functionality of its domains, we used the CRISPR/Cas9 genome editing method to create a series of *PWL1* mutants in the JG30 background. At the beginning, we chose a target close to the Gly412Arg amino acid substitution site and obtained two different types of homozygous editing plants (*ko‐1* and *ko‐2*) containing a single nucleotide insertion, which retain the SP, B‐lectin, SLG, and PAN domains but lack the TM and STK domains (Figure S3c,e). As excepted, both *ko‐1* and *ko‐2* showed withered leaves and whitish leaf tips at the three‐leaf stage (Figure 2d) and began to rapidly wither and then died at the four‐leaf stage (Figure 2e). To analyse whether the TM and STK domains of PWL1 are necessary for rice survival, we designed two targets at the C‐terminus of PWL1 and generated another two different types of homozygous knockout mutants (*ko‐3* and *ko‐4*). The *ko‐3* mutant had a single nucleotide insertion at target 2, which lacks the STK domain. The *ko‐4* mutant had a 2 bp deletion at target 3, resulting in a frameshift mutation and only 78 amino acids different from the WT protein (Figure S3d,e). In a surprise, both *ko‐3* and *ko‐4* exhibited withered leaves at the three‐leaf stage (Figure 2f), and the *ko‐3* mutant died at the four‐leaf stage, while *ko‐4* grew longer to the five‐leaf stage but with severe retardation and eventually died (Figure 2g). Overall, these results revealed that LOC_Os03g62180 is the *PWL1* gene, which is essential for rice growth, and disruption of key structure domains can lead to seedling lethality.

Based on a BLASTP search using the PWL1 sequence as the query, a total of 22 PWL1 homologous proteins (more than 50% identity with PWL1) were identified from monocot and dicot plant species. Phylogenetic analysis showed that PWL1 homologues are ubiquitous in various monocots and dicots, whereas monocot orthologs showed higher sequence similarity (Figure S4c). Multiple sequence alignment of PWL1 homologues among rice, *Panicum hallii*, *Zea mays*, *Sorghum bicolor*, *Brachypodium distachyon*, *Hordeum vulgare*, *Triticum dicoccoides*, *Gossypium hirsutum*, and *Arabidopsis thaliana* revealed that these PWL1 homologous proteins have a highly conserved protein kinase domain (Figure S5).

### Expression of *PWL1* and protein properties of PWL1

Quantitative real‐time PCR (qRT‐PCR) was performed to explore the expression profile of *PWL1*. We found that *PWL1* was constitutively expressed in various rice tissues tested, including roots, culms, leaves, sheath, and panicle, at the seedling, tillering and heading stages. The expression level of *PWL1* was relatively higher in leaves than in other tissues (Figure S6a). To understand the transcriptional level of *PWL1* during leaf ageing, we measured the *PWL1* expression in young leaves, early‐senescing leaves, and late‐senescence leaves of the JG30 plants. The expression of *PWL1* was higher in the young leaves but lower in the late‐senescence leaves (Figure S6b), indicating that *PWL1* was not induced by leaf senescence. Then we analysed whether the Gly412Arg amino acid substitution would affect gene expression and found that the mRNA level of *pwl1* was similar to that of the WT (Figure S6c).

Based on SoftBerry ProtComp 9.0 (http://linux1.softberry.com/), PWL1 was predicted to be localized in the plasma membrane. To further verify the subcellular localization of PWL1, we fused the full‐length CDSs of *PWL1* and *pwl1* to the N‐terminus of green fluorescent protein (GFP) under the control of the CaMV 35S promoter. The PWL1‐GFP and pwl1‐GFP fusion proteins and the plasma membrane marker PIP2‐mCherry (Heng *et al*., 2018) were co‐transfected into protoplasts of rice and *Nicotiana benthamiana*, respectively. GFP fluorescence observation revealed that the PWL1‐GFP and pwl1‐GFP fusion proteins were completely colocalized with the plasma membrane marker signal (Figure 3e and Figure S7a), suggesting that PWL1 is a plasma membrane protein, and the Gly412Arg substitution did not alter the subcellular localization of the pwl1 protein.

Moreover, the bimolecular fluorescence complementation (BiFC) assay revealed that PWL1 could self‐associate (Figure 3f). Previous studies report the PAN domain as a major mediator of receptor dimerization, self‐interaction activity, and the formation of two dimer interfaces (Naithani *et al*., 2007). Therefore, to investigate whether the PAN structure in PWL1 can self‐interact, we conducted research on the PN fragment harbouring the PAN domain (aa 329–434) of PWL1 and pwl1. The yeast two‐hybrid (Y2H) assay revealed that both PWL1‐PN and pwl1‐PN exhibited self‐interaction activity (Figure 3g). Whereas the BS fragment harbouring the B‐lectin domain and SLG domain (aa 36–310) showed relatively weak self‐interaction activity, and the C‐terminal fragment containing the kinase domain did not exhibit autoactivation (Figure S7b,c). We further validated the self‐interaction of PN fragments in *vivo* using co‐immunoprecipitation (Co‐IP) assays with rice protoplasts. The results showed that PWL1‐PN‐Flag and pwl1‐PN‐Flag were co‐immunoprecipitated when PWL1‐PN‐GFP and pwl1‐PN‐GFP were pulled down from protoplast extracts with anti‐GFP beads, respectively (Figure 3h). Notably, when pwl1‐PN‐Flag and pwl1‐PN‐GFP were co‐introduced into rice protoplasts, the abundance of pwl1‐PN‐Flag protein was significantly reduced after co‐immunoprecipitation (Figure 3h and Figure S7d,e), suggesting that the Gly412Arg amino acid substitution in pwl1 may have affected the ability of PAN to interoperate with itself.

### Mutation of *PWL1* leads to enhanced ROS accumulation and cell death in rice leaves

**Figure 4:**
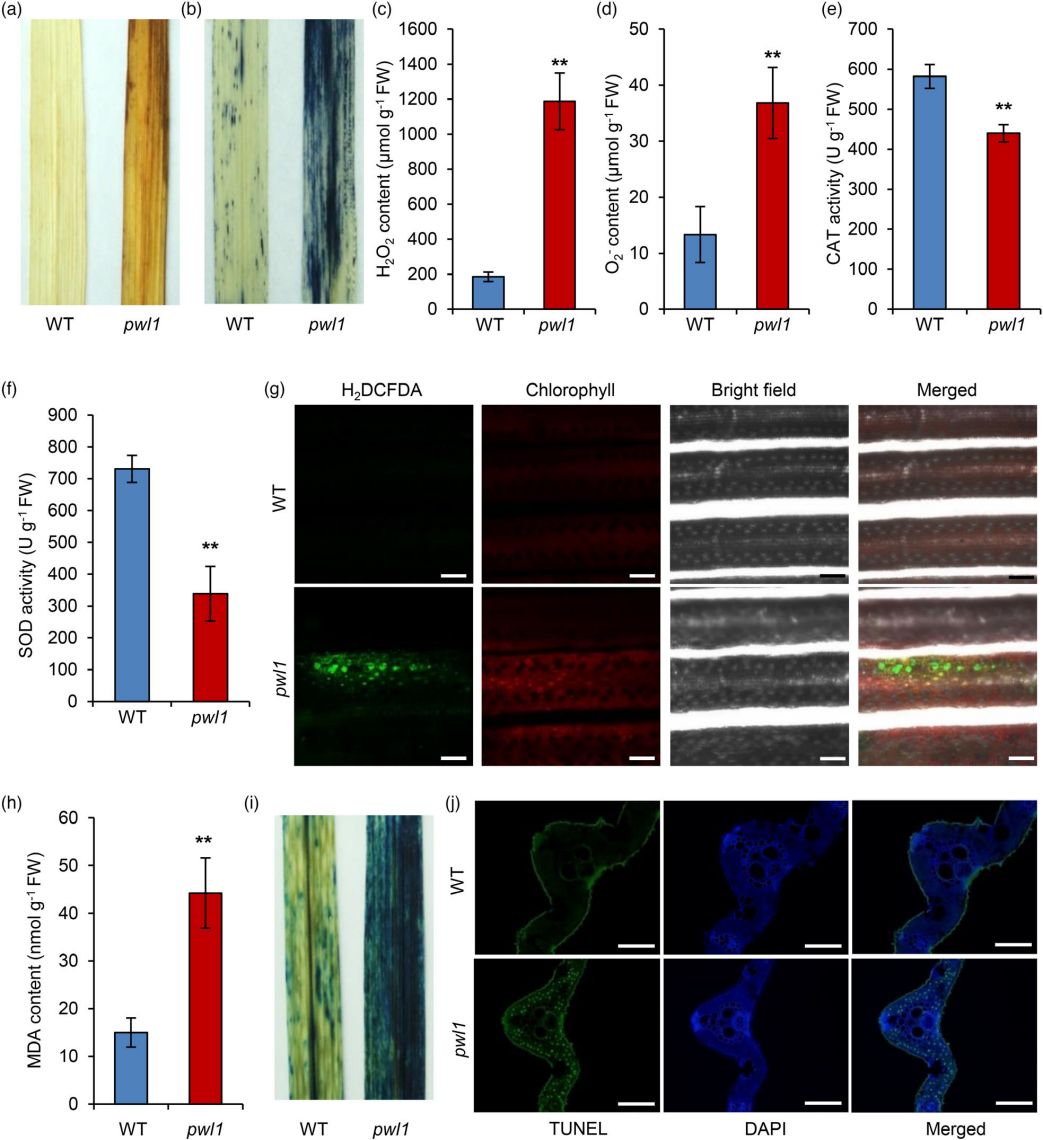
Reactive oxygen species (ROS) accumulation and premature cell death in the *pwl1* leaves. (a) Visible detection of H_2_O_2_ in wild type (WT) and *pwl1* by DAB staining. (b) Visible detection of O_2_^−^ in WT and *pwl1* by NBT staining. (c–f) Measurement of H_2_O_2_ content (c), O_2_^−^ content (d), CAT activity (e), and SOD activity (f) in WT and *pwl1* plants. (g) H_2_DCFDA (ROS‐indicating) fluorescence of mesophyll cells from leaves of WT and *pwl1* plants. Green indicates oxidized H_2_DCFDA and red indicates chlorophyll. Bars, 80 μm. (h) Measurement of MDA contents in WT and *pwl1* plants. (i) Visualization of cell death in WT and *pwl1* by trypan blue staining. (j) TUNEL assay of WT and *pwl1* leaves at the tillering stage. The blue signal is 4′6‐diamino‐phenylindole (DAPI) staining, and the green fluorescence represents TUNEL‐positive signals. Bars, 50 μm. Data are means ± SD (*n* = 6). **P < 0.01 (Student's *t*‐test).

Abnormal ROS accumulation can cause cell oxidation, damage chloroplast development, and trigger cell death, resulting in accelerated leaf senescence (Kim *et al*., 2016; Lim *et al*., 2007). Therefore, we used several different histochemical staining methods to detect the level of ROS in the leaves at the tillering stage in *pwl1* plants. DAB staining showed deeper reddish brown in the *pwl1* leaves while there was no obvious staining in the WT leaves, indicating increased H_2_O_2_ accumulation in the *pwl1* leaves (Figure 4a). NBT staining showed densely blue spots in the *pwl1* leaves compared with those of WT leaves, suggesting excessive accumulation of O_2_^−^ (Figure 4b). Consistently, the concentrations of H_2_O_2_ and O_2_^−^ were significantly higher in the *pwl1* leaves compared to the WT (Figure 4c,d). Conversely, the activities of antioxidant enzymes catalase (CAT) and superoxide dismutase (SOD) were remarkably decreased in *pwl1* than in WT (Figure 4e,f). We determined the cellular ROS production using the specific fluorescent dye 2′,7′‐dichlorodihydrofluorescein diacetate (H_2_DCFDA). Confocal microscopy showed green fluorescence in the *pwl1* leaves but not in the WT leaves (Figure 4g). Furthermore, we analysed the concentration of the lipid oxidation product malondialdehyde (MDA) and found that the *pwl1* leaves have a higher level of MDA than the WT leaves (Figure 4h). These outcomes showed that the *pwl1* leaves had an excessive accumulation of ROS.

The premature leaf senescence in the *pwl1* mutant suggested that accelerated programmed cell death (PCD) had occurred. We performed trypan blue staining and the terminal deoxynucleotidyl transferase dUTP nick‐end labelling (TUNEL) assay to determine cell death. Trypan blue staining revealed densely deep‐blue spots on *pwl1* leaves, while little positive blue staining was observed on WT leaves (Figure 4i). A hallmark of PCD is the condensation of nuclear chromatin, caused by DNA fragmentation in the nucleus. The TUNEL assay can sensitively detect DNA fragmentation. We accordingly carried out the TUNEL assay to detect apoptotic cell death in the *pwl1* leaves. As expected, more and stronger green positive fluorescence signals were observed in the *pwl1* leaf cells, whereas nearly no TUNEL signals were observed in the WT leaves (Figure 4j and Figure S8), indicating that severe DNA damage occurred in the *pwl1* leaves. These data show that mutations in *PWL1* result in premature cell death.

### Mutations in *PWL1* affect the expression of genes associated with multiple biological processes

To reveal the molecular basis for the regulation of PWL1, we performed a yeast two‐hybrid (Y2H) screen of a rice cDNA library using the C‐terminus of PWL1 (amino acids 460–859) as the bait. However, we were unable to obtain any positive‐interacting clones. Thus, to investigate the biological processes interfered with by the *PWL1* mutation, we performed RNA sequencing (RNA‐seq) to profile the transcriptomes of WT and *pwl1*. Correlation analysis and volcano plot analysis revealed that the transcriptome profiles of *pwl1* were significantly different from those of WT (Figure S9a,b). Overall, 3875 DEGs were identified (*P*‐value <0.05), with 3125 genes significantly up‐regulated and 750 genes down‐regulated in *pwl1* (Figure S9c). To explore the biological processes associated with these DEGs, we carried out gene ontology (GO) enrichment analysis and showed that metabolic processes, catabolic processes, protein folding and translation were enriched in *pwl1* (Figure S9d). We also performed Kyoto Encyclopedia of Genes and Genomes (KEGG) enrichment analysis and revealed that these DEGs are involved mainly in the ribosome, carbon metabolism, peroxisome, and cytochrome P450 (Figure S9e).

**Figure 5:**
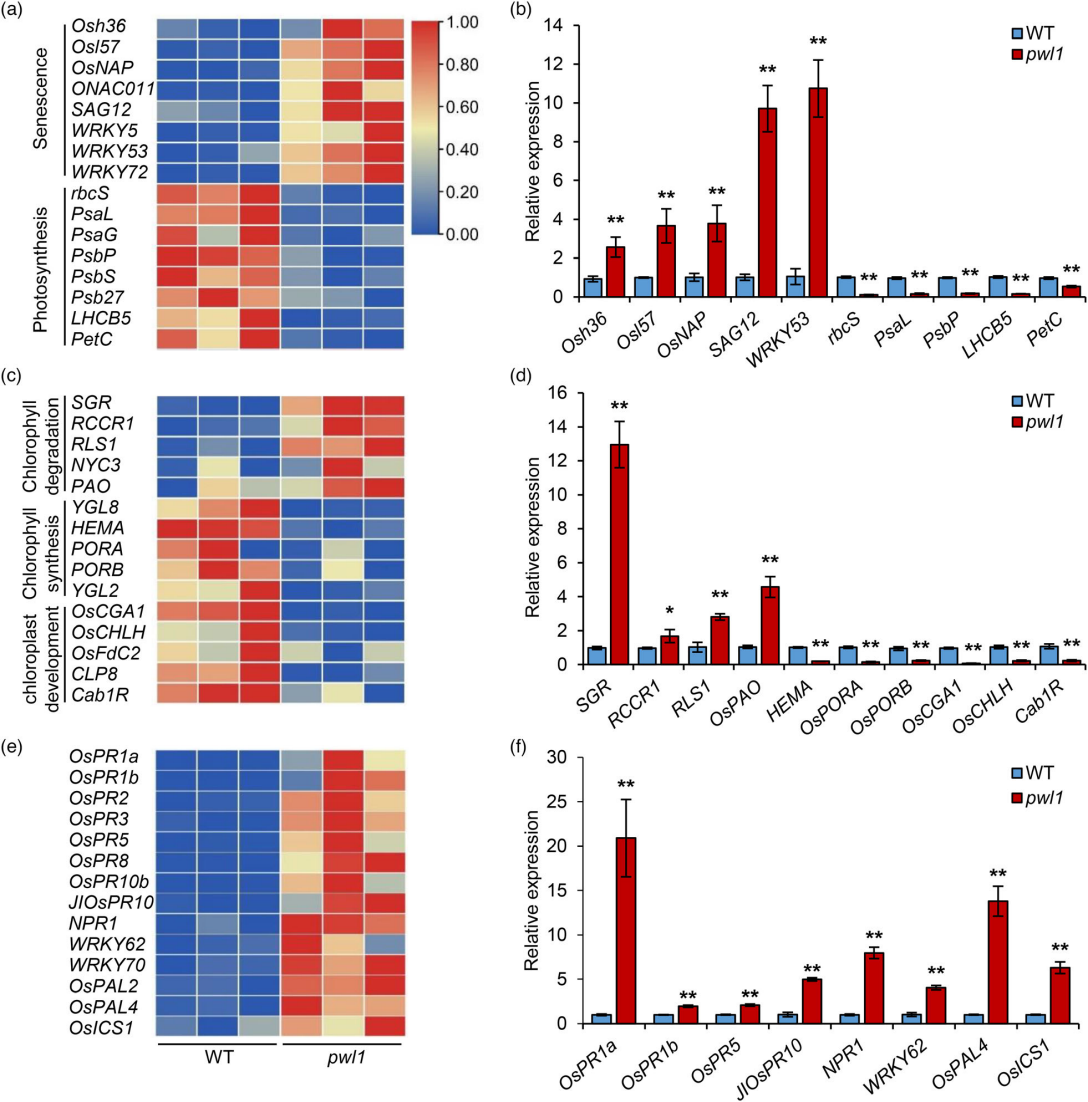
Transcriptome analysis of leaves in the *pwl1* mutant compared with the wild type (WT). (a) Heat map of senescence and photosynthesis‐related gene expression. Three biological replicates were performed. (b) qRT‐PCR validation of several senescence‐ and photosynthesis‐related genes in (a). (c) Heat map of chlorophyll degradation, chlorophyll synthesis, chlorophyll development‐related gene expression. (d) qRT‐PCR validation of several chlorophyll degradation, chlorophyll synthesis, and chlorophyll development‐related genes in (c). (e) Heat map of defence‐related gene expression. (f) qRT‐PCR validation of several defence‐related genes in (e). Rice *OsActin1* was used as an internal control. Data are means ± SD (*n* = 3). *P < 0.05; **P < 0.01 (Student's *t*‐test).

In the RNA‐seq database, we first examined the expression of SAGs in *pwl1* and WT plants. We found that some SAGs, such as *Osh36*, *Osl57*, *OsNAP*, *SAG12*, and *WRKY5* were up‐regulated in the *pwl1* mutant (Figure 5a). While the expression levels of the genes involved in photosynthesis were significantly declined in the *pwl1* compared to the WT. Meanwhile, we also found that the expression profiles of these SAGs were consistent with the RNA‐seq database through qRT‐PCR (Figure 5b). Given the decrease of chlorophyll and abnormal chloroplast development in *pwl1*, we next analysed whether the genes involved in chlorophyll degradation, chlorophyll synthesis and chloroplast development were differentially expressed in *pwl1* compared to the WT. We found that some DEGs related to chlorophyll degradation, such as *SGR*, *RLS1*, and *OsPAO*, which promote chlorophyll degradation, were up‐regulated in *pwl1* (Figure 5c). However, the transcript abundances of the genes responsible for chlorophyll synthesis and chloroplast development were down‐regulated in *pwl1* (Figure 5c). Likewise, we also used qRT‐PCR to examine the expression of these genes, and the results are consistent with the RNA‐seq data (Figure 5d). These results suggest that *PWL1* may directly or indirectly regulate the expression profiles of several genes related to senescence, photosynthesis, chloroplast development and chlorophyll synthesis.

### The premature leaf senescence phenotype of the *pwl1* mutant is induced by heat stress

**Figure 6:**
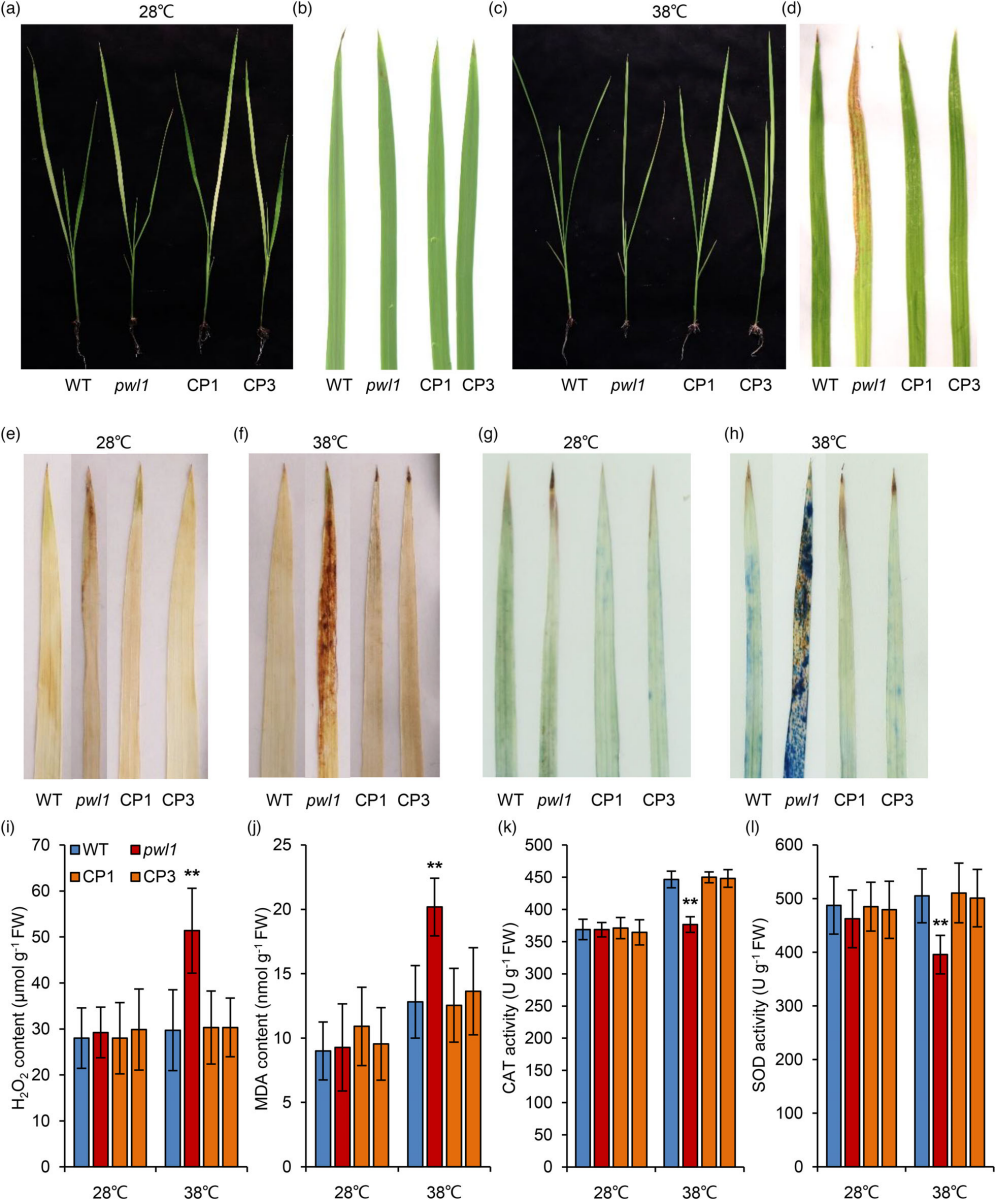
Effects of high‐temperature treatment on wild type (WT) and *pwl1* at the seedling stage. (a) Phenotypic comparison of WT, *pwl1*, CP1, and CP3 seedlings under normal temperature treatment. (b) Phenotype of the second leaf in (a). (c) Phenotypic comparison of WT, *pwl1*, CP1, and CP3 seedlings under high‐temperature treatment. (d) Phenotype of the second leaf in (c). (e, f) DAB staining in leaves of WT, *pwl1*, CP1, and CP3 at 28 °C (e) and 38 °C (f). (g, h) Trypan blue staining in leaves of WT, *pwl1*, CP1, and CP3 at 28 °C (g) and 38 °C (h). (i–l) Measurement of H_2_O_2_ content (i), MDA content (j), CAT activity (k), and SOD activity (l) in WT, *pwl1*, CP1, and CP3 plants under different temperature conditions. Data are means ± SD (*n* = 6). **P < 0.01 (Student's *t*‐test).

Based on the GO enrichment analysis, we further found that response to temperature stimulus and response to heat were in the enriched biological processes (Figure S9d), implying that *PWL1* might be associated with heat stress. To test this hypothesis, we analysed the high‐temperature stress among WT, *pwl1*, and complementary transgenic lines (CP1 and CP3). Seedlings were cultured in growth chambers for 14 days under normal (28 °C) or high (38 °C) temperature conditions. Under normal temperature conditions, the *pwl1* seedlings were not significantly different from the WT, CP1, and CP3 plants; only a few lesions occurred on the older leaf tips of *pwl1* (Figure 6a,b). However, after high‐temperature treatment, the *pwl1* had more severe necrosis and was filled with larger and more dense lesions, while no noticeable lesions were found in the WT, CP1, and CP3 plants (Figure 6c,d). Subsequently, we observed the phenotypic differences between WT and *pwl1* under severe high‐temperature stress. Two‐week‐old seedlings grown at 28 °C were transferred into a growth chamber at 40 °C for 7 day, followed by a recovery culture at 28 °C for 7 day, and then the survival rate of the seedlings was evaluated. We found that *pwl1* showed severe heat stress sensitivity and most of the leaves withered (Figure S10a), with a survival rate of 17.5%, while the survival rate of the WT was as high as 87.5% (Figure S10b). We concluded that the *pwl1* mutant was more sensitive to high‐temperature stress.

Then we investigated whether *PWL1* participated in controlling ROS production at high temperatures. Staining with DAB showed no significant difference in H_2_O_2_ contents in the WT, *pwl1*, CP1, and CP3 plants under normal temperature conditions (Figure 6e), whereas more H_2_O_2_ contents were detected in the *pwl1* leaves compared to the WT and complementary transgenic lines after the seedlings had been exposed to higher temperatures (Figure 6f). We then detect the amount of ROS by applying the specific fluorescent probe H_2_DCFDA. No fluorescence signals were detected in all seedlings under normal temperature conditions (Figure S11a), but the signals were denser in *pwl1* after high‐temperature treatment, and no signals were found in the WT and complementary transgenic lines (Figure S11b). Similar results were obtained for trypan blue staining, meaning that more cell death occurred in *pwl1* after high‐temperature treatment (Figure 6g,h). Likewise, the contents of H_2_O_2_ and MDA were significantly higher than in the *pwl1* than the WT and complementary transgenic lines under at high temperatures (Figure 6i,j). Additionally, the activities of antioxidant enzymes CAT and SOD were significantly reduced in the *pwl1* compared to the WT and complementary transgenic lines at high temperatures (Figure 6k,l). These results indicated that hyper‐accumulation of ROS in *pwl1* might trigger the PCD, thereby triggering the occurrence of lesion and wilting leaves after high‐temperature treatment.

We also used TEM to determine the chloroplast ultrastructure in the WT, *pwl1*, and CP1 plants under normal and high‐temperature conditions. In normal temperature conditions, the chloroplasts showed no obvious differences among the WT, *pwl1*, and CP1 plants, with intact chloroplast and thylakoid structures and dense grana lamellae (Figure S12a). Differently, the *pwl1* leaves showed defective chloroplasts compared to the WT and CP1 leaves at high‐temperature conditions. The *pwl1* mutant had a few irregular chloroplasts, more degraded chloroplasts and impaired thylakoid development compared to the WT and CP1 leaves (Figure S12b), indicating that the high temperature induces chloroplast degradation in the *pwl1* mutant. Taken together, the above results suggest that *PWL1* plays an important role in heat tolerance in rice.

### Mutations in *PWL1* confer rice resistance to bacterial pathogens

**Figure 7:**
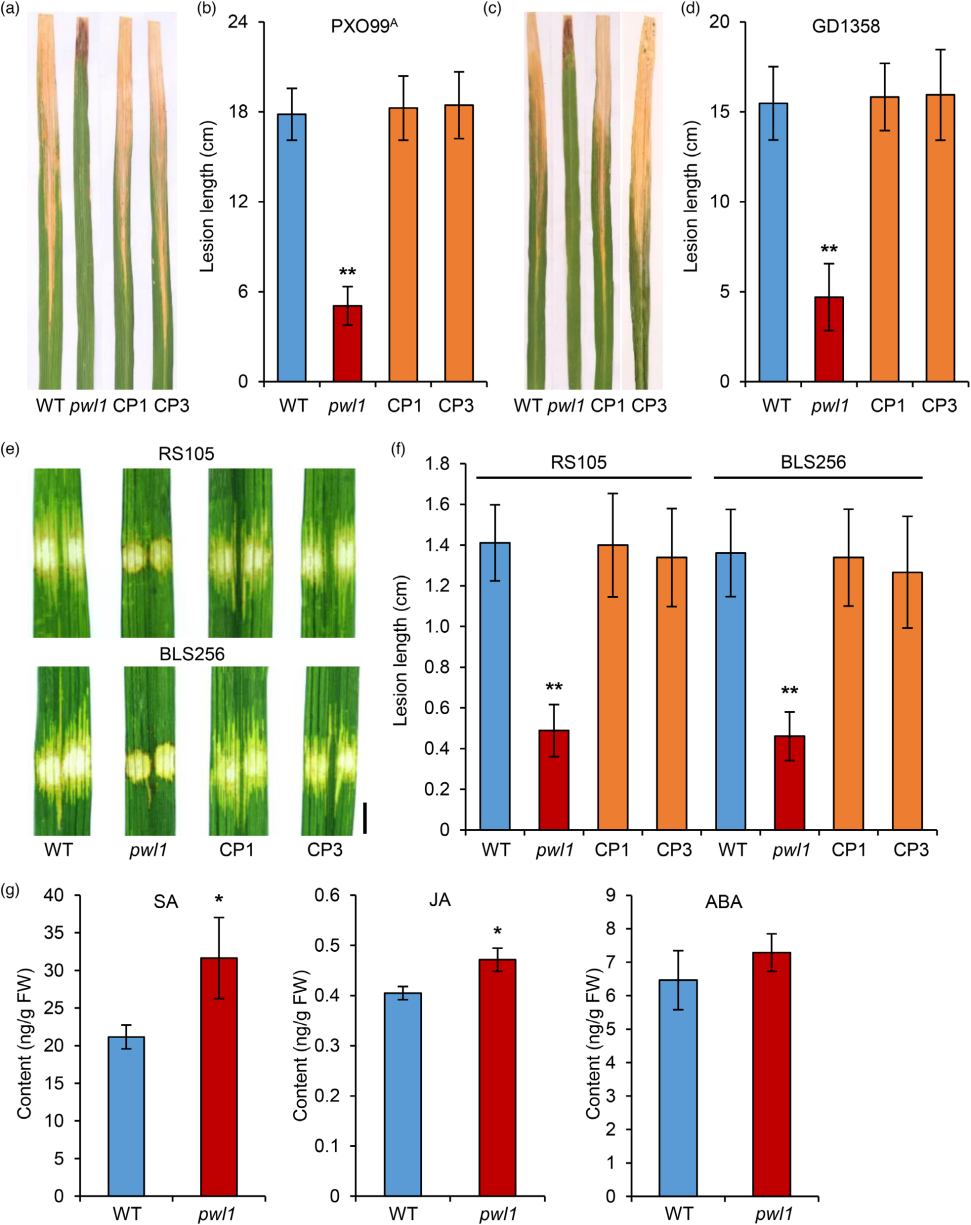
The *pwl1* mutant displays enhanced resistance to *Xoo* and *Xoc*. (a) Leaves of wild type (WT), *pwl1*, CP1, and CP3 plants were inoculated with *Xoo* strain PXO99^A^. (b) Lesion lengths were measured 14 days after inoculation with PXO99^A^. Values are mean ± SD (*n* = 6). ** *P* < 0.01 (Student's *t*‐test). (c) Leaves of WT, *pwl1*, CP1, and CP3 plants were inoculated with *Xoo* strain GD1358. (d) Lesion lengths were measured 14 days after inoculation with GD1358. Data are means ± SD (*n* = 6). ** *P* < 0.01 (Student's *t*‐test). (e) Leaves of WT, *pwl1*, CP1, and CP3 plants were inoculated with *Xoc* strains RS105 and BLS256. Bar, 0.5 cm. (f) Lesion lengths were measured 5 days after inoculation with RS105 and BLS256. Values are mean ± SD (*n* = 6). **P < 0.01 (Student's *t*‐test). (g) Content of salicylic acid (SA), jasmonic acid (JA), and abscisic acid (ABA) in WT and *pwl1* plants. Data are means ± SD (*n* = 3). *P < 0.05 (Student's *t*‐test).

Since *pwl1* had hypersensitive response‐like lesions and excessive accumulation of ROS, we speculated that *pwl1* might exhibit enhanced rice disease resistance. Then, we inoculated the WT, *pwl1*, CP1, and CP3 plants with the *Xoo* strains PXO99^A^ and GD1358 at the tillering stage. Two weeks post‐inoculation, the lesion lengths of *pwl1* were shorter than those of WT, CP1, and CP3 plants (Figure 7a–d), indicating that *pwl1* confers enhanced resistance to *Xoo*. Then, we also inoculated the representative *Xoc* strains RS105 and BLS256 at the tillering stage. Five days post‐inoculation, the *pwl1* mutant showed increased resistance to *Xoc* strains compared with WT, CP1, and CP3 plants, as shown by analyses of the lesion length (Figure 7e,f). To further investigate the mechanism of enhanced resistance in *pwl1*, we examined the expression of pathogenesis‐related (PR) genes in *pwl1* and WT plants from the RNA‐seq database. Interestingly, we found that many PR genes involved in salicylic acid (SA) and JA signalling pathways were significantly up‐regulated in *pwl1* (Figure 5e). Meanwhile, we also validated the expression profiles of several selected genes through qRT‐PCR, and the results were consistent with the RNA‐seq database; the expression levels of *OsPR1a* and *OsPAL4* were more than 10‐fold higher in *pwl1* than in WT (Figure 5f). Furthermore, the content of SA and JA is higher in *pwl1* than in WT (Figure 7g). These results suggest that the Gly412Arg amino acid substitution in *pwl1* confers enhanced resistance to the bacterial pathogens *Xoo* and *Xoc*, possibly by activating the expression of several disease resistance‐related genes in rice.

### Haplotype and nucleotide diversity analysis of *PWL1*

**Figure 8:**
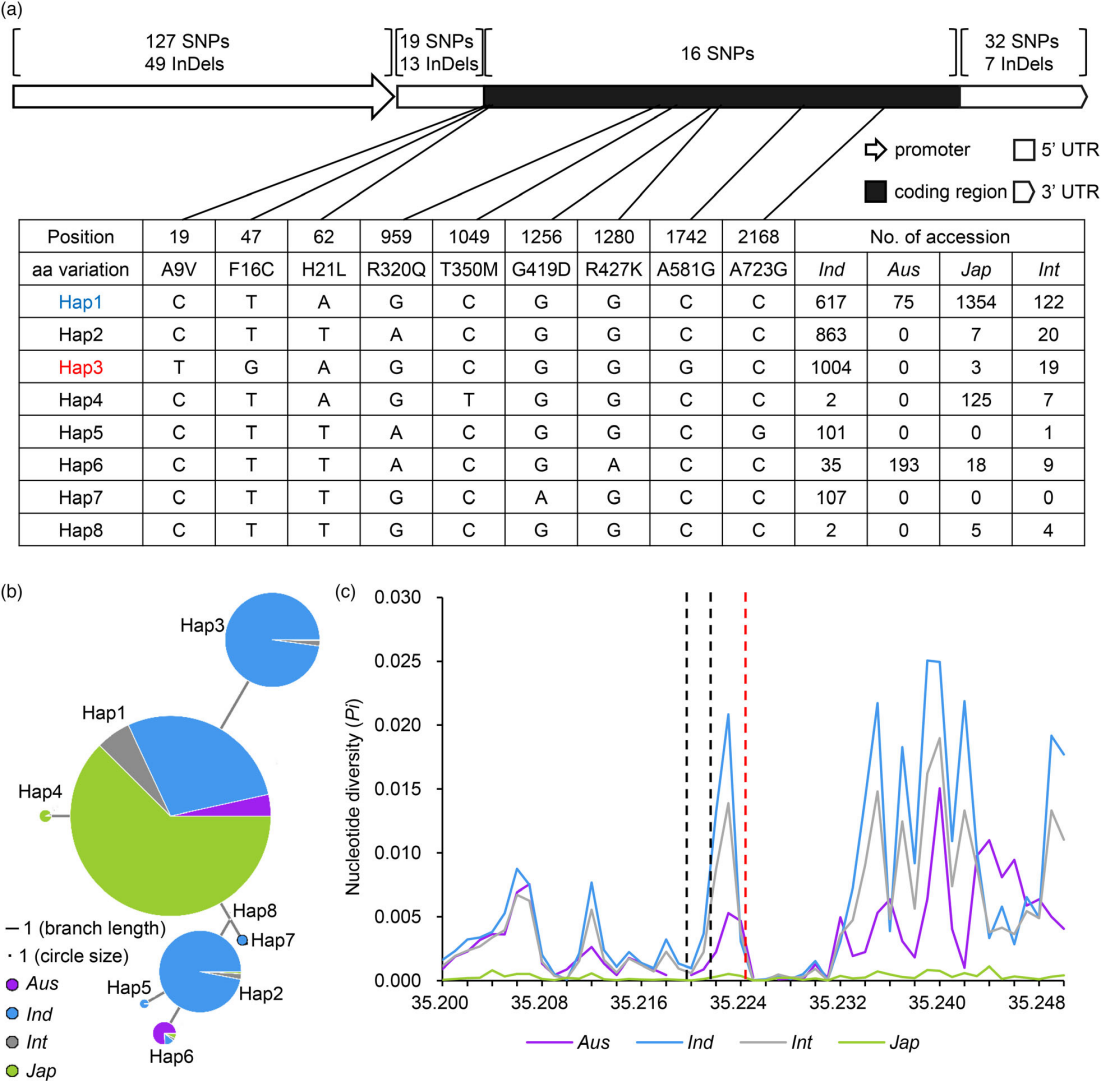
The natural variation analysis of *PWL1*. (a) Haplotype analysis of 4726 rice accessions. The blue font Hap1 indicates the reference (Nipponbare) allele sequence, and the red font Hap3 indicates the allele sequence of JG30. (b) Haplotype network of *PWL1*. The size of circles is proportional to the number of accessions for a given haplotype, and their frequencies for different types of germplasm are shown by colour. (c) Nucleotide diversity of *Pi* for different subgroups in the 200‐kb region spanning *PWL1*. The two black dashed lines represent the coding region, and the red dashed line on the right represents the promoter region. Hap, haplotype; aa, amino acid; *Ind*, *indica* subpopulation; *Aus*, *aus* subpopulation; *Jap*, *japonica* subpopulation; *Int*, *intermediate* subpopulation.

To search for natural alleles of *PWL1*, we performed haplotype analysis of *PWL1* among 4726 rice accessions using the RiceVarMap v.2.0 database (http://ricevarmap.ncpgr.cn/). The rice accessions included *indica* (*Ind*), *aus* (*Aus*), *japonica* (*Jap*), and *intermediate* (*Int*). We found only 16 single‐nucleotide polymorphisms (SNPs) in the CDS region of *PWL1*. By contrast, the 2‐kb promoter sequence possessed 127 SNPs and 49 InDels, the 5′ untranslated region (UTR) sequence had 19 SNPs and 13 InDels, and the 3′ UTR sequence had 32 SNPs and 7 InDels (Figure 8a). Excluding low‐frequency variations and nonsense mutations, eight major haplotypes (Hap 1–8) were classified by nine SNPs, but no variant type of Gly412Arg was found (Figure 8a,b). The Hap1 accessions were identified in all four subspecies, containing 617 *indica*, 75 *aus*, 1354 *japonica*, and 122 *intermediate* cultivars. The Hap2, Hap3, and Hap5 accessions were mainly identified in *indica*, whereas the Hap4 accessions were mainly identified in *japonica*. And the JG30 belongs to Hap3. The Hap6 accessions were mainly identified in *aus*, containing 193 *aus*, 35 *indica*, 18 *japonica*, and 9 *intermediate* cultivars. The Hap7 accessions were only identified in *indica*, including 107 accessions. The Hap8 accessions were only identified in 11 cultivars, containing 2 *indica*, 5 *japonica*, and 4 *intermediate* cultivars. Nucleotide diversity (*Pi*) analysis of the 200‐kb region spanning the *PWL1* gene indicated that *indica* has higher *Pi* values than that of *japonica* subspecies, and the average *Pi* values of the CDS region were lower than those in the promoter region (Figure 8c), suggesting that PWL1 in *japonica* cultivars was under stronger selection during domestication and breeding and that the PWL1 protein is relatively conserved.

## Discussion

### *PWL1* encodes a functional lectin receptor kinase protein required for leaf senescence and plant growth in rice

LecRLKs, a subset of the LRKs family, participate in biotic/abiotic stress responses and plant development (Bellande *et al*., 2017; Sun *et al*., 2020). However, the molecular mechanism by which LecRLKs regulate plant leaf senescence is unknown. In this study, we isolated a rice mutant, *pwl1*, which exhibited natural leaf withering and premature leaf senescence. Map‐based cloning revealed that *PWL1* encodes a G‐type LecRLK (Figure 2), and the *pwl1* mutant was caused by a single amino acid substitution (Gly412Arg) in PWL1. The *pwl1* plant showed leaf withering from the seedling stage, and the symptoms became aggravated along with growth, and the leaves were basically wilted at the booting stage (Figure 1a–c). Like most premature leaf senescence mutants (Chen *et al*., 2021; Leng *et al*., 2017; Xu *et al*., 2022a), the premature leaf senescence of *pwl1* has a negative impact on its agronomic traits, with only 50% seed setting rate and a significant reduction in the plant height, tiller number, and 1000‐grain weight (Table S1). Genetic complementarity experiments proved that *PWL1* could rescue the premature leaf senescence phenotype of *pwl1*, including normal leaf senescence and agronomic traits (Figure 2 and Figure S3). Surprisingly, knockout of *PWL1* resulted in seedling lethality (Figure 2d–g). We generated a series of *PWL1* mutants with truncation of different domains by using the CRISPR/Cas9 method. The edited lines *ko‐1* and *ko‐2* lacked the TM and STK domains, and the edited line *ko‐3* lacked the STK domain; all of them showed seedling lethality at the three‐leaf stage. In contrast, the edited line *ko‐4* lacked only part of the C‐terminal amino acids; it survived longer and died until the reproductive growth stage. Haplotype analysis revealed that the mutation of Gly412Arg is unique in pwl1 and that the mutation can maintain plant growth and seed setting (Figure 8a), presumably eliminating the mutation by natural selection due to adverse effects on plant growth. These results suggest that PWL1 plays crucial roles in leaf senescence and plant growth in rice.

PWL1 has the characteristic domain of G‐type LecRLKs, featuring an extracellular B‐lectin domain, an SLG, a PAN, a TM, and an intracellular STK domain (Sun *et al*., 2020). The B‐lectin domain contains a β‐barrel structure and shows potential binding affinity to α‐D mannose, but its specific function is still unknown. Previous studies have reported that mutations in a glycosyltransferase gene, *PLS2*, result in a phenotype similar to *pwl1* and significant sucrose accumulation (Wang *et al*., 2018). In the RNA‐seq data, the expression profile of *PLS2* in the wild‐type was similar to that of *pwl1*, suggesting that *PWL1* may regulate the binding of D‐mannose through other regulatory pathways. Furthermore, the STK domain of PWL1 contains all the necessary conserved features for a functional kinase (Figure S5). It is emphasized that the kinase activity of LecRLKs is crucial for gene function through phosphorylation events (Sun *et al*., 2020). For example, Peng *et al*. (2020) reported that the kinase activity was required for an L‐type LecRLK OsLecRK‐S.7 function in regulating pollen development and plant growth in rice. He *et al*. (2021b) revealed an L‐type LecRLK AP1 in controlling pollen maturity of rice. AP1 interacted with and phosphorylated OsUGP2, a pollen‐preferential protein that plays key roles in pollen starch biosynthesis and pollen maturation. It wassuggested that the kinase activity may be required for maintaining the normal enzymatic activity of OsUGP2. Our results showed that the kinase domain of PWL1 was an active kinase with autophosphorylation activities, and the K524E mutation lost its kinase activity (Figure 3b). Yet, how PWL1 initiates and transmits developmental signals to downstream components to regulate leaf senescence remains unclear. Our RNA‐seq analysis showed that genes promoting senescence were up‐regulated, whereas those genes involved in photosynthesis and chloroplast development were down‐regulated in *pwl1* (Figure 5a–d). The small molecule metabolic process, oxidoreductase activity, carboxylic acid metabolic process, and cytoplasmic part were enriched in the DEGs, inferring that PWL1 may possibly affect the above biological processes and related genes. We also used the rice yeast library to screen for downstream substrates and did not detect the interacting proteins, presumably due to the low abundance of the interacting proteins. Therefore, future studies can identify the signalling molecules that trigger PWL1 and the signalling cascade response downstream of PWL1.

### Mutations in *PWL1* promote ROS accumulation and enhanced disease resistance in rice

Plant leaf senescence is one of the developmental processes controlled by environmental signals, in which ROS play an active role in promoting leaf senescence (Domínguez and Cejudo, 2021). ROS are toxic substances that can cause the oxidation of DNA, RNA, proteins, lipids, and many kinds of small molecules in cells (Mittler, 2017; Mittler *et al*., 2022). We detected high levels of ROS in *pwl1* leaves by DAB and NBT staining (Figure 4a,b), H_2_DCFDA staining (Figure 4g), and measurement of the contents of H_2_O_2_ and O_2_^−^ (Figure 4c,d). ROS homeostasis in plant cells is usually determined by the balance between ROS generation and ROS scavenging (Mittler *et al*., 2022). ROS are mainly produced in apoplast by NADPH oxidases (termed respiratory burst oxidase homologues, RBOHs), in chloroplast, mitochondria, and peroxisome through different pathways (Mignolet‐Spruyt *et al*., 2016). Based on our KEGG enrichment analysis, many DEGs were significantly enriched in peroxisome pathway (Figure S9e). Moreover, RNA‐seq data showed three RBOH genes, *OsrbohB*, *OsrbohE*, and *Osrboh8* were up‐regulated (Table S7), implying more ROS production in the *pwl1* mutant. ROS scavenging mainly includes CAT, SOD, peroxidase (POD), and ascorbate peroxidase (APX) (Mittler, 2017). We showed that the activities of CAT and SOD were significantly reduced in the *pwl1* mutant (Figure 4e,f). These findings suggest that *PWL1* is important for maintaining ROS homeostasis. Meanwhile, leaf senescence is accompanied by PCD caused by hyperaccumulation of ROS, a mechanism that has been elucidated in rice premature leaf senescence mutants (Mittler, 2017). The *ypd1* (*yellow and premature dwarf 1*) mutant showed premature senescence and accelerated cell death due to higher ROS levels in the leaves (Chen *et al*., 2021). The *hpa1* mutant exhibited H_2_O_2_ accumulation and PCD, subsequently triggering leaf senescence (Xiong *et al*., 2021). The *oswss1* (*water‐soaked spot1*) mutant exhibited hyper‐accumulation of ROS, more severe DNA fragmentation, and cell death (Xu *et al*., 2022a). In this study, the *pwl1* mutant displayed typical PCD features, including cell death and severe DNA fragmentation (Figure 4i,j). Together, we speculated that *PWL1* is involved in leaf senescence via ROS‐mediated enhanced PCD.

ROS play a central role in plant innate immunity, and the accumulation of ROS and the appearance of cell death are major phenotypes associated with enhanced disease resistance (Qi *et al*., 2017). Consistently, in the present study, the *pwl1* mutant displayed markedly enhanced resistance to the bacterial pathogens *Xoo* and *Xoc* (Figure 7a–f), and the expression of PR genes was activated in *pwl1* (Figure 5e,f), such as *OsPR1a*, *OsPR1b*, *NPR1*, and *WRKY62*, which are known to be involved in the SA signalling pathway, and *JIOsPR10* and *OsPR10b* in the JA signalling pathway (Liu *et al*., 2017; Zhang *et al*., 2023). Furthermore, the contents of endogenous SA and JA in *pwl1* were higher than those in WT plants (Figure 7g), suggesting that mutation of *PWL1* led to the activation of defence‐related genes and enhanced disease resistance in rice. It is well known that SA and JA positively regulate senescence and disease resistance in plants, and crosstalk exists between ROS and SA (Wang *et al*., 2020b; Woo *et al*., 2019). In *Arabidopsis*, it is reported that the transcription factor WRKY55 positively regulates leaf senescence and pathogen resistance by directly modulating the transcription of genes implicated in the biosynthesis of SA and ROS (Wang *et al*., 2020b). Here, the *pwl1* mutant showed premature leaf senescence and resistance to bacterial pathogens through ROS, SA, and JA accumulation. Thus, future studies can determine whether PWL1 also involved in regulating leaf senescence and disease resistance by regulating the expression of ROS‐, SA‐, and JA‐related genes.

### *PWL1* plays essential roles in heat tolerance via modulating ROS homeostasis and chloroplast function

Temperature plays an important role in regulating the growth and development of crops (Ding *et al*., 2020). Global warming seriously threatens crop growth and productivity, thereby increasing the risk of food insecurity (Lobell *et al*., 2011). In this study, we showed that the *pwl1* mutant is more sensitive to high temperatures at the seedling stage. Compared with the WT, *pwl1* exhibited a heat‐sensitive phenotype when grown at a higher temperature (38 °C), with severe necrosis and withered leaves (Figure 6c,d). Moreover, *pwl1* was more susceptible and had a lower survival rate than WT when treated at 40 °C (Figure S10). Studies have shown that heat stress is the main environmental factor causing premature leaf senescence in plants. The *premature senescence leaf 50* (*psl50*) mutant showed heat‐induced premature leaf senescence in rice (He *et al*., 2021a). The rice *high‐temperature enhanced lesion spots 1* (*hes1*) mutant displayed more severe lesion‐mimic symptoms and leaf withering after high‐temperature treatment (Xia *et al*., 2022). Similarly, we found that premature leaf senescence of *pwl1* was accelerated by higher temperatures in the field at 1‐month late sowing, and *pwl1* appeared to have rapid premature leaf senescence in the tillering stage (Figure S13). Overall, our data showed that high temperatures can promote premature leaf senescence in *pwl1*.

Along with temperature increases, ROS accumulates in the plant, but hyper‐accumulation of ROS can lead to oxidative stress and cell death (Mittler *et al*., 2022). Indeed, we detected high levels of ROS in *pwl1* plants under high‐temperature conditions (Figure 6e,f,i). Similarly, *pwl1* has higher levels of cell death at high temperatures compared with the WT and complementary transgenic lines (Figure 6g,h). In addition, we found ROS‐scavenging enzymes such as CAT and SOD were significantly lower than those in WT and the complementary transgenic lines under high‐temperature conditions (Figure 6k,l). In plants, chloroplasts are factories for metabolic intermediates and act as a hub to sense environmental signals, such as high temperatures (Mittler *et al*., 2022; Zhang *et al*., 2022). Therefore, we observed the chloroplast ultrastructure under normal and high‐temperature conditions. It was shown that the chloroplast structures of WT, *pwl1*, and CP1 displayed no significant difference at normal temperature conditions (Figure S12a). However, at high‐temperature conditions, the *pwl1* mutant showed disrupted chloroplasts with distorted and impaired lamellae and appeared to have more degraded chloroplasts compared with the WT and CP1 plants (Figure S12b). Thus, we proposed that *PWL1* may respond to high temperatures by affecting ROS homeostasis and chloroplast function.

### The putative molecular mechanism of *PWL1* in rice

RLKs respond to external ligands by forming homodimerization or heterodimerization, followed by activation of downstream signalling factors by the intracellular kinase domains via phosphorylation (Osakabe *et al*., 2013). Here, we found that a G‐type LecRLK PWL1 could self‐association, similar to previous reports that a L‐type LecRLK OsLecRK5 and a RLK STRK1 can self‐association in rice (Wang *et al*., 2020a; Zhou *et al*., 2018). A G‐type LecRLK contains a PAN domain, which is characterized by the presence of two disulfide bonds linking the alpha‐helix to the central region of the protein (Tordai *et al*., 1999). The PAN motif plays key roles in protein–protein or protein–carbohydrate interactions and facilitates receptor dimerization (Naithani *et al*., 2007). In this study, the Gly412Arg substitution disrupts the PAN module in the *pwl1* mutant (Figure 3d), suggesting that its function is impaired. Furthermore, the Y2H and Co‐IP assays showed that the PAN module of PWL1 interacted with itself (Figure 3g,h), indicating that the PAN plays an important role in the self‐interaction of PWL1. Interestingly, the ability of the PAN to interact with itself is diminished in pwl1 (Figure 3h). A previous study revealed that the PAN domain acts as the catalytic core of signalling and determines the fate of downstream signalling cascades; PAN mutations can decrease MAPK phosphorylation and phosphorylation of its downstream targets (Pal *et al*., 2022). Thus, we speculate that the Gly412Arg amino acid substitution in pwl1 reduces its ability to interact with itself, followed by impaired activation of downstream targets, resulting in impaired function of downstream signalling.

**Figure 9:**
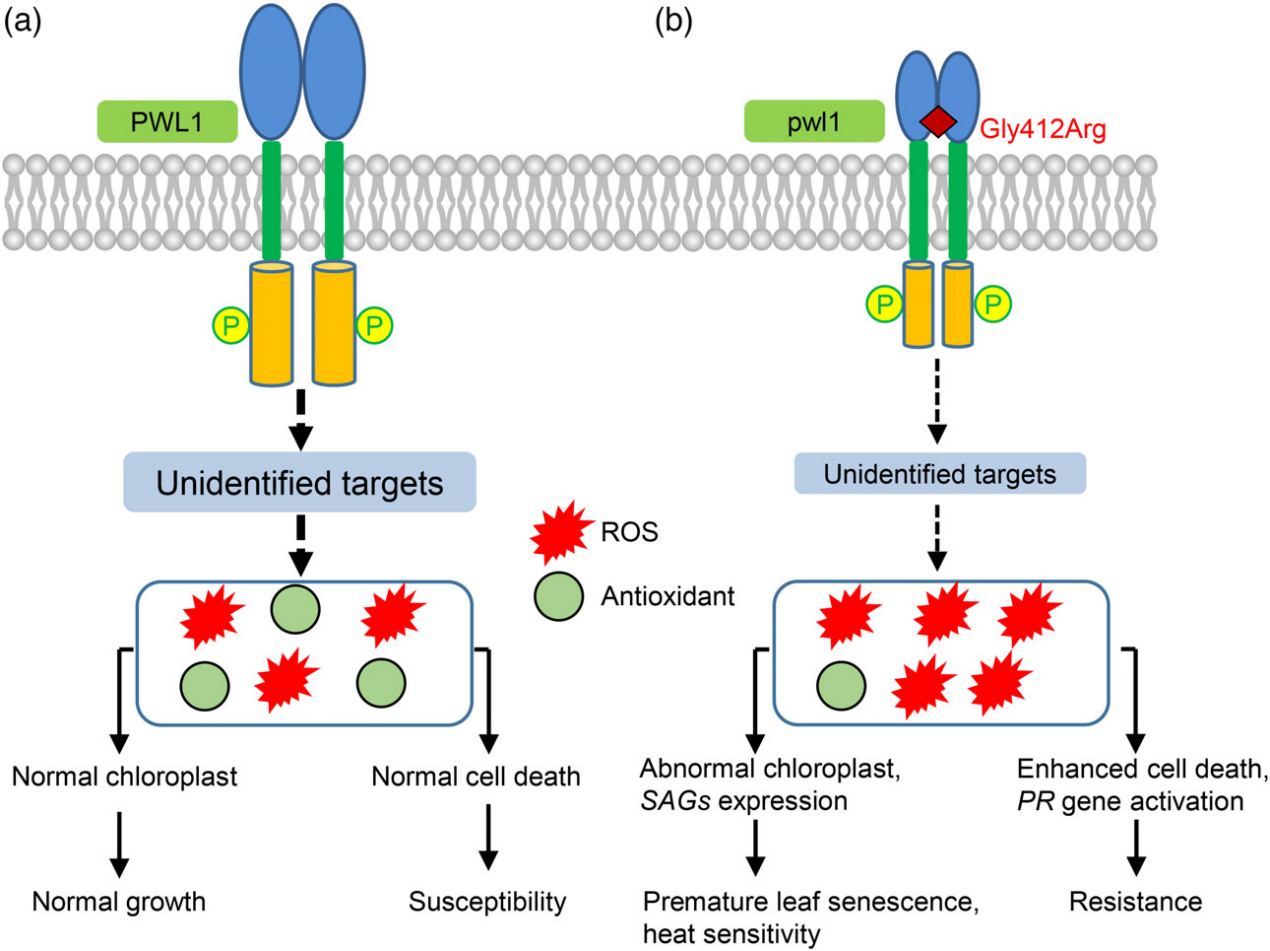
A proposed model illustrates the function of PWL1 in rice. (a) PWL1 can interact with itself, activate downstream unidentified target proteins, and subsequently maintain normal ROS homeostasis, normal chlorophyll development, cell death, and the normal growth and susceptibility to disease of rice plant. (b) In the *pwl1* mutant, the Gly412Arg substitution of PWL1 impaired self‐association and had reduced activation (depicted by the reduced size of the dimer), resulting in impaired activation or reduced activity of downstream signalling, causing ROS hyper‐accumulation, triggering abnormal chloroplast development, induced the expression of *SAGs* and *PR* genes, and enhanced cell death, ultimately resulting in premature leaf senescence, heat sensitivity and enhanced resistance to bacterial pathogens. The thick arrows indicate normal function, and the thin arrows indicate depressed function.

We concluded that PWL1 positively regulates leaf senescence and heat tolerance but negatively regulates resistance to bacterial pathogens by maintaining ROS homeostasis and PCD in rice (Figure 9). In the WT, PWL1 can self‐associate, activate downstream unidentified target proteins, and subsequently maintain normal ROS homeostasis, normal chloroplast development, and cell death. However, in the *pwl1* mutant, pwl1 is impaired in self‐association and has reduced activation, causing impaired activation or reduced activity of downstream signalling, which leads to ROS accumulation, triggering abnormal chloroplast development, induced expression of *SAGs* and *PR* genes, and enhanced cell death, ultimately exhibiting the phenotypes of premature leaf senescence, heat sensitivity, and enhanced resistance to bacterial pathogens.

## Materials and methods

### Plant materials and growth conditions

The *pwl1* mutant was isolated from an ethyl methanesulfonate (EMS)‐mutagenized library of the *indica* rice cultivar Jingang 30 (JG30). Rice plants were grown in the paddy fields at the experimental stations in Beijing and Hainan province, China, under natural conditions. Yellowing seedlings for protoplast isolation were grown in a growth chamber at 37 °C under continuous darkness.

The high‐temperature growth stress treatment was performed as described previously (Xia *et al*., 2022). Briefly, rice seeds were germinated and grown in a growth chamber (14 h : 10 h, light:dark photoperiod) at 28 °C and 38 °C for 14 day. For heat treatment, rice plants were grown in a growth chamber under a photoperiod of 14/10 h light/dark at 28 °C for 2 weeks, then transferred to a high temperature of 40 °C for 7 day, followed by a 7‐day recovery at 28 °C, and the survival rate of seedlings was evaluated.

For dark treatment of plants at the seedling stage, 2‐week‐old seedlings (wild type and *pwl1*) were transferred into a growth chamber at 28 °C under continuous darkness for 6 day, followed by a 7‐day recovery under a photoperiod of 14/10 h light/dark at 28 °C, after which the survival rate of seedlings was evaluated.

For the dark treatment of detached leaves, the flag leaves of 1‐month‐old rice plants (wild type and *pwl1*) were detached and floated on ddH_2_O in complete darkness at 28 °C for 5 day.

### Measurement of chlorophyll concentrations

The concentrations of chlorophyll a (Chl *a*), chlorophyll b (Chl *b*), and carotenoid (Car) were determined as described in our previous research (Xu *et al*., 2022a). Briefly, leaves of wild type and *pwl1* were detached and immersed in an 80% acetone (v/v) solution for 48 h at 4 °C in the dark. The sample solution was measured at 665, 649, and 470 nm using a BioPhotometer Plus (Eppendorf, Germany). The 80% acetone was used as a blank control. The chlorophyll concentrations were calculated following a published method (Lichtenthaler, 1987). There are three biological replicates for each group.

### Transmission electron microscopy

For transmission electron microscopy analysis of the chloroplast ultrastructure, the leaf samples were collected and fixed in a 2.5% pre‐cooled glutaraldehyde solution at 4 °C for 2 days. Subsequent procedures were performed following a published protocol (Zheng *et al*., 2021) and then observed with a Hitachi HT7700 transmission electron microscope.

### Map‐based cloning of *PWL1*

The *pwl1* mutant was crossed with the *japonica* cultivar Nipponbare (NIP) and the *indica* cultivar JG30 for genetic analysis. A total of 725 F_2_ individuals with mutant phenotypes from the NIP/*pwl1* cross were selected for DNA extraction and gene mapping. *PWL1* was roughly mapped to an interval between two insertion/deletion (InDel) markers, ID3‐17 and ID3‐22, near the telomere of Chromosome 3. For fine mapping, new InDel markers were developed based on genome polymorphisms between NIP and JG30. Mutation sites within candidate genes were detected through direct sequencing of the PCR‐amplified genomic DNA from wild‐type and *pwl1* mutants. Primers used for mapping are shown in Table S3.

### Vector construction and genetic transformation

For genomic DNA complementation, a 4.9 kb genomic DNA fragment containing the wild‐type *PWL1* promoter as well as its coding region was amplified by PCR using primers pPWL1‐CF and pPWL1‐CR (Table S5). The amplified fragment was cloned into the binary vector pCAMBIA1300 using the ClonExpress®II One‐Step Cloning Kit (Vazyme, China). The resulting construct, pCAMBIA1300‐pPWL1::PWL1, was transformed into *pwl1* calli via *Agrobacterium tumefaciens*‐mediated transformation, and the plants were regenerated.

For CRISPR/Cas9‐mediated gene knockout mutation, a 20‐bp gene‐specific guide RNA sequence near the mutation site of *pwl1* was synthesized, annealed, and ligated into pYLsgRNA‐U6a, which was subcloned into the vector pYLCRISPR/Cas9Pubi‐H (Ma *et al*., 2015). Two target gRNA sequences on the C‐terminal structural domain of PWL1 were designed and inserted into the vector pYLCRISPR/Cas9Pubi‐H. The constructs were transformed into the embryogenic calli derived from JG30 by *Agrobacterium tumefaciens*‐mediated transformation. PCR‐based sequencing was performed to verify the mutation sites of the target gene. All primer sequences are listed in Table S5.

### RNA extraction and quantitative real‐time PCR

Total RNAs from different tissues were extracted using the RNAiso Plus reagent (Takara, Japan) according to the manufacturer's manual. A total of 1 μg RNA was reversely transcribed into complementary DNA (cDNA) using the HiScript III 1st Strand cDNA Synthesis Kit (+gDNA wiper) (Vazyme, China). Quantitative real‐time PCR (qRT‐PCR) was conducted as described in our previous research (Zheng *et al*., 2021). Briefly, qRT‐PCR reactions were performed on an ABI 7500 Real Time PCR system (Life Technologies, Carlsbad, CA, USA) with a Taq Pro Universal SYBR qPCR Master Mix (Vazyme, China), according to the manufacturer's instructions. Rice *OsActin1* (*LOC_Os03g50885*) was used as an internal control. The relative expression analysis was calculated by the 2^−ΔΔCT^ method (Livak and Schmittgen, 2001). Primers used for qRT‐PCR are listed in Table S6.

### Subcellular localization of PWL1

The coding regions of *PWL1* and *pwl1* in wild‐type and mutant plants were amplified using primers PWL1‐GFP‐F and PWL1‐GFP‐R (Table S5). All fragments were inserted into the p35S::GFP vector and transformed into protoplasts derived from the leaf sheaths of 14‐day‐old JG30 seedlings for transient expression. Additionally, the constructs were transiently expressed in *Nicotiana benthamiana* leaves by *Agrobacterium tumefaciens*‐mediated infiltration (Gao *et al*., 2016). Green fluorescent protein (GFP) fluorescence signals were observed using a Zeiss LSM700 laser scanning confocal microscope.

### Histochemical assays

The 3,3′‐diaminobenzidine (DAB) and nitroblue tetrazolium (NBT) stainings were performed as described previously (Wu *et al*., 2017). Briefly, leaves were soaked in 0.1% (w/v) DAB (Sigma) or 0.05% (w/v) NBT (Duchefa) solutions, followed by incubation at 37 °C for 16 h under dark conditions. The stained leaves were transferred into 95% ethanol to bleach out the chlorophyll, and then transferred to 70% glycerol for photographing. Trypan blue staining was used to detect dead cells according to the method of a published protocol (Zheng *et al*., 2021). Briefly, leaves were submerged in a lactic acid‐phenol‐Trypan blue solution (25% lactic acid, 23% water‐saturated phenol, 0.25% Trypan blue, and 25% glycerol) and boiled for 10 min. After being kept in the dark overnight, the stained leaf samples were incubated in 25 mg/mL chloral hydrate to decolorize for 3 days. The blue spots on the leaves were photographed.

The 2′,7′‐dichlorofluorescein diacetate (H_2_DCFDA) experiment was used to determine cellular ROS production according to the method of a published protocol (Hu *et al*., 2021).

### Measurement of various reactive oxygen species‐related indexes

The contents of H_2_O_2_ and MDA and the activities of SOD and CAT in leaf samples were measured using the kits (A064‐1‐1, A003‐1‐2, A001‐1‐2, and A007‐1‐1) following the manufacturer's manuals (Nanjing Jiancheng Bioengineering Institute, China). The content of O_2_^−^ in leaf samples was determined using a kit (R30343) following the manufacturer's instructions (Shanghai Yuanye Bio‐Technology, China). Briefly, at the tillering stage, fresh leaves (0.2 g) were collected and ground into homogenate with 1.8 mL of 50 mmol L^−1^ precooled sodium phosphate buffer (pH 7.8). After centrifuging for 10 min at 12 000 ***g*** at 4 °C, the supernatant was collected for determination of the contents of H_2_O_2_, O_2_^−^, and MDA, and the activities of SOD and CAT.

### TUNEL assay

The TUNEL assay was performed as described in our previous research using the Fluorescein *In Situ* Cell Death Detection Kit (Roche) (Xu *et al*., 2022a). Briefly, leaves of wild type and *pwl1* were fixed in FAA solution for 24 h, then embedded in paraffin and cut into thin slices. The paraffin was eluted with ethanol series, and the slices were stained with 4′,6‐diamidino‐2‐phenylindole (DAPI). The TUNEL signal was observed using a Zeiss LSM700 laser scanning confocal microscope.

### Pathogen inoculation

Three to five fully expanded rice leaves per plant were inoculated with *X. oryzae* pv. *oryzae* (*Xoo*) strains by the leaf‐clipping method and *X. oryzae* pv. *oryzicola* (*Xoc*) by infiltration using a needleless syringe as described previously at the tillering stage (Ji *et al*., 2016). The *Xoo* strains used in this study included one Philippine strain, PXO99^A^, and one Chinese strain, GD1358. The *Xoc* strains used in this study included RS105 and BLS256. Disease symptoms were photographed and measured 14 days after *Xoo* inoculation and 5 days after *Xoc* inoculation, respectively. Each *Xoo* and *Xoc* strain was inoculated on 5–10 rice plants.

### Measurement of phytohormones in rice leaves

The quantification of endogenous SA, JA, and ABA was performed as described previously (Liu *et al*., 2023). Briefly, flag leaves from 1‐month‐old plants were collected. After grinding in liquid nitrogen, about 200 mg of powder from each sample was extracted in methanol containing 200 ng of ^2^H_4_‐SA, D_6_‐JA, and ^6^H‐ABA used as internal standards for SA, JA, and ABA, respectively, and shaken in the dark for at least 2 h at 4 °C. Following centrifugation, the supernatant was collected, evaporated to dryness under a nitrogen gas stream, and re‐solubilized in 100 μL of 30% methanol. The extracts were analysed using an LC–MS/MS system (EXPEC 5210). Three independent biological samples were analysed.

### RNA‐seq analysis

The leaves of wild type and *pwl1* were collected at the tillering stage, and total RNA was extracted using TRIzol reagent (Invitrogen). Three biological replicates of wild type and *pwl1* were used for RNA‐seq analysis. RNA‐seq analysis was performed on an Illumina Xten platform at Shanghai Sangon Biotech Co., Ltd. Raw data (Raw reads) were processed by Trimmomatic and mapped to the *Indica* ShuHui498 reference genome (http://www.mbkbase.org/rice) using HISAT2. The read counts of each gene were calculated with StringTie. Differentially expressed genes (DEGs) were detected through the DESeq2 R package with the thresholds of log_2_ fold change >2 and <−2 (*q*‐value < 0.05). GO analysis was performed with the topGO R package. KEGG pathway enrichment was assigned using the clusterProfiler package.

### Phosphorylation assay

The coding sequences of the intracellular domain of PWL1 were amplified using primers His‐PWL1‐IF and His‐PWL1‐IR (Table S5) and cloned into pET28a for fusing in‐frame with the 6 × His tag. The point mutation construct PWL1^K524E^‐C was generated by PCR‐based site‐directed mutagenesis. The fusion constructs were transformed into *Escherichia coli* expression strain *Rosetta* (DE3) (Solarbio). The protein expression was induced with 0.2 mm isopropyl β‐D‐thiogalactoside (IPTG) for 20 h at 18 °C. Bacterial cells were collected and affinity‐purified using the High Affinity Ni‐NTA Resin (L00250; GenScript Biotech Corp., Piscataway, NJ, USA) following the manufacturer's instructions. For the assay of PWL1 autophosphorylation, 1 mg of purified recombinant His‐PWL1‐C and His‐PWL1^K524E^‐C was incubated in kinase reaction buffer (50 mm Tris–HCl, pH 7.5, 10 mm MgCl_2_, 1 mm DTT, and 1 mm ATP) for 30 min at 30 °C. The reaction was stopped with the addition of SDS‐PAGE loading buffer, and the phosphorylation of PWL1 was evaluated by immunoblotting analysis using an antiphospho (Ser/Thr) Phe antibody (Abcam, ab17464).

### Protein interaction assay

For the bimolecular fluorescence complementation (BiFC) assay, the full‐length coding sequence of *PWL1* was inserted into both pSPYNE and pSPYCE vectors. These constructs were transformed into *Agrobacterium* strain GV3101, and BiFC assays were conducted by a previously described method (Waadt and Kudla, 2008). The YFP signal was detected using the Zeiss LSM700 laser scanning confocal microscope.

The yeast two‐hybrid (Y2H) assays were performed following the Matchmaker system (Clontech). The PN fragment harbouring the PAN domain (amino acids 329–434) of PWL1 and pwl1, the BS fragment harbouring the B‐lectin and SLG domains (amino acids 36–310), and the intracellular domain of PWL1 (amino acids 460–859) were amplified by PCR and then inserted into both pGBKT7 and pGADT7 vectors. The resulting pGBKT7 and pGADT7 constructs were then co‐transformed into yeast strain AH109. All the transformed cells were grown on synthetically defined (SD) medium lacking leucine and tryptophan (−LW) and SD medium lacking leucine, tryptophan, histidine, and adenine (−LWHA).

For Co‐immunoprecipitation assay (Co‐IP) in rice protoplasts, the PN fragment (amino acids 329–434) of PWL1 and pwl1 were amplified and cloned into both pCAMBIA1300‐35S‐GFP and pCAMBIA1300‐35S‐Flag vectors. GFP + PWL1‐PN‐Flag, or PWL1‐PN‐GFP + PWL1‐PN‐Flag, or pwl1‐PN‐GFP + pwl1‐PN‐Flag were transfected into rice protoplasts. Total protein was extracted with extraction buffer (50 mmol/L Tris–HCl, pH 7.5, 150 mmol/L NaCl, 2% Triton X‐100, 20% glycerol, 1 mmol/L EDTA, 1 mmol/L phenylmethylsulfonyl fluoride (PMSF), and 1 × protease inhibitor cocktail). The proteins were then incubated with 15 μL of prewashed anti‐GFP magnetic beads (MBL, Japan) for 1 h at 4 °C with rotation. The beads were washed three times with wash buffer (50 mmol/L Tris–HCl, pH 7.5, 150 mmol/L NaCl, 0.1% Triton X‐100, 20% glycerol, 1 mmol/L EDTA, 1 mmol/L PMSF) at 4 °C for 15 min, and resuspended with 2 × SDS loading buffer and boiled for 10 min. The samples were then separated onto 10% SDS‐PAGE gels and subjected to Western blot analysis with anti‐FLAG (MBL, Japan) and anti‐GFP antibodies (MBL, Japan). The protein quantification was calculated using Image J software. All primer sequences for the constructs are listed in Table S5.

### Bioinformatics analysis and haplotype analysis

The full‐length PWL1 protein sequence (BAS87274) was used as a query for identifying its homologues proteins through the public Basic Local Alignment Search Tool from the National Center for Biotechnology Information database (http://www.ebi.ac.uk/Tools/sss/ncbiblast/). Multiple protein alignments were performed using the DNAMAN program. The phylogenetic trees were constructed using MAGE 7.0 version software using the bootstrap method with 1000 bootstrap replicates. Conserved domains of the PWL1‐encoded proteins were analysed using SMART (http://smart.embl‐heidelberg.de/). The three‐dimensional structures were prepared with PyMol (https://pymol.org/).

The haplotypes of *PWL1* were analysed using the genotype data of 4726 rice accessions downloaded from the RiceVarMap v.2.0 database (http://ricevarmap.ncpgr.cn/) (Zhao *et al*., 2021). Nucleotide diversity (*Pi*) of a 200‐kb region containing *PWL1* was calculated using VCFtools with a 1000‐bp window method and 1000‐bp step size (Danecek *et al*., 2011).

## Author contributions

ZJ and KZ designed the study. JX, CW, FW, YL, ML, HW, and YZ conducted fieldwork and laboratory analyses. XJ, KZ, and ZJ wrote the first drafts of the manuscript and all authors contributed to later versions of the manuscript and to data interpretation.

## Competing interests

None declared.

## Supporting information


**Figure S1** Comparison of internode length and pollen grains between wild type (WT) and *pwl1*.
**Figure S2** Dark stress‐induced senescence phenotype of *pwl1* mutant.
**Figure S3** Molecular identification of complementary transgenic lines and edited rice plants.
**Figure S4** Kinase activity and phylogenetic tree of PWL1.
**Figure S5** Protein sequence alignment of PWL1 and its homologues from several species.
**Figure S6** Expression pattern of *PWL1*.
**Figure S7** The protein properties of the PWL1 protein.
**Figure S8** Comparison of the numbers of TUNEL‐positive cells in the wild type (WT) and *pwl1* mutant.
**Figure S9** Transcriptome analysis, gene ontology (GO) and Kyoto Encyclopedia of Genes and Genomes (KEGG) analysis of DEGs in wild type (WT) and *pwl1*.
**Figure S10** The rice mutant *pwl1* was more sensitive to heat stress.
**Figure S11** Representative images of H_2_DCFDA fluorescence from mesophyll cells from leaves of wild‐type (WT) and *pwl1* plants measured at 28 °C (a) and 38 °C (b).
**Figure S12** Ultrastructure of chloroplasts in mesophyll cells of the wild type (WT), *pwl1* and CP1 at 28 °C (a) and 38 °C (b).
**Figure S13** Comparison of gross morphology between wild‐type (WT) and *pwl1* plants in the paddy field at 1‐month late sowing.
**Table S1** Comparison of major agronomic traits among the wild‐type (WT), *pwl1* and the PWL1 complemented plants (CP1 and CP3).
**Table S2** Genetic analysis of the *pwl1* mutant.
**Table S3** Markers used for fine mapping.
**Table S4.** List of open reading frames in the 85.54‐kb target region.
**Table S5** Primers used for vector construction and transgenic line test.
**Table S6** Primers sequences related to quantitative real‐time PCR.
**Table S7** Differentially expressed of SAGs, photosynthesis, chloroplast metabolism and ROS generation‐related genes in *pwl1* and wild‐type (WT) plants.
