# Supplementary material for: PWL1, a G‐type lectin receptor‐like kinase, positively regulates leaf senescence and heat tolerance but negatively regulates resistance to Xanthomonas oryzae in rice
Source: Plant Biotechnol J. 2023 Aug 14;21(12):2525–45. doi: 10.1111/pbi.14150 (PMC10651159; doi:10.1111/pbi.14150)
Supplement: Supplementary file 1 — Figure S1 Comparison of internode length and pollen grains between wild type (WT) and pwl1. Figure S2 Dark stress‐induced senescence phenotype of pwl1 mutant. Figure S3 Molecular identification of complementary transgenic lines and edited rice plants. Figure S4 Kinase activity and phylogenetic tree of PWL1. Figure S5 Protein sequence alignment of PWL1 and its homologues from several species. Figure S6 Expression pattern of PWL1. Figure S7 The protein properties of the PWL1 protein. Figure S8 Comparison of the numbers of TUNEL‐positive cells in the wild type (WT) and pwl1 mutant. Figure S9 Transcriptome analysis, gene ontology (GO) and Kyoto Encyclopedia of Genes and Genomes (KEGG) analysis of DEGs in wild type (WT) and pwl1. Figure S10 The rice mutant pwl1 was more sensitive to heat stress. Figure S11 Representative images of H2DCFDA fluorescence from mesophyll cells from leaves of wild‐type (WT) and pwl1 plants measured at 28 °C (a) and 38 °C (b). Figure S12 Ultrastructure of chloroplasts in mesophyll cells of the wild type (WT), pwl1 and CP1 at 28 °C (a) and 38 °C (b). Figure S13 Comparison of gross morphology between wild‐type (WT) and pwl1 plants in the paddy field at 1‐month late sowing. Table S1 Comparison of major agronomic traits among the wild‐type (WT), pwl1 and the PWL1 complemented plants (CP1 and CP3). Table S2 Genetic analysis of the pwl1 mutant. Table S3 Markers used for fine mapping. Table S4. List of open reading frames in the 85.54‐kb target region. Table S5 Primers used for vector construction and transgenic line test. Table S6 Primers sequences related to quantitative real‐time PCR. Table S7 Differentially expressed of SAGs, photosynthesis, chloroplast metabolism and ROS generation‐related genes in pwl1 and wild‐type (WT) plants. [file PBI-21-2525-s001.zip › Supplementary Figures S1-S13+Tables S1-S7(1).docx]

**PWL1, a G-type lectin receptor-like kinase, positively regulates leaf senescence and heat tolerance but negatively regulates resistance to *Xanthomonas* *oryzae* in rice**

Jiangmin Xu^1†^, Chunlian Wang^1†^, Fujun Wang^1,2^, Yapei Liu^1^, Man Li^1^, Hongjie Wang^1^, Yuhan Zheng^1^, Kaijun Zhao^1^*, Zhiyuan Ji^1^*

^1^*National Key Facility for Crop Gene Resources and Genetic Improvement, Institute of Crop Sciences, Chinese Academy of Agricultural Sciences, Beijing, China*

^2^*Institute of Rice Research, Guangdong Academy of Agricultural Sciences, Guangzhou, China*

**Correspondence* (Tel 86-10-82108751; emails jizhiyuan@caas.cn (Z.J.); zhaokaijun@caas.cn (K.Z.))

^†^These authors contributed equally to this work.


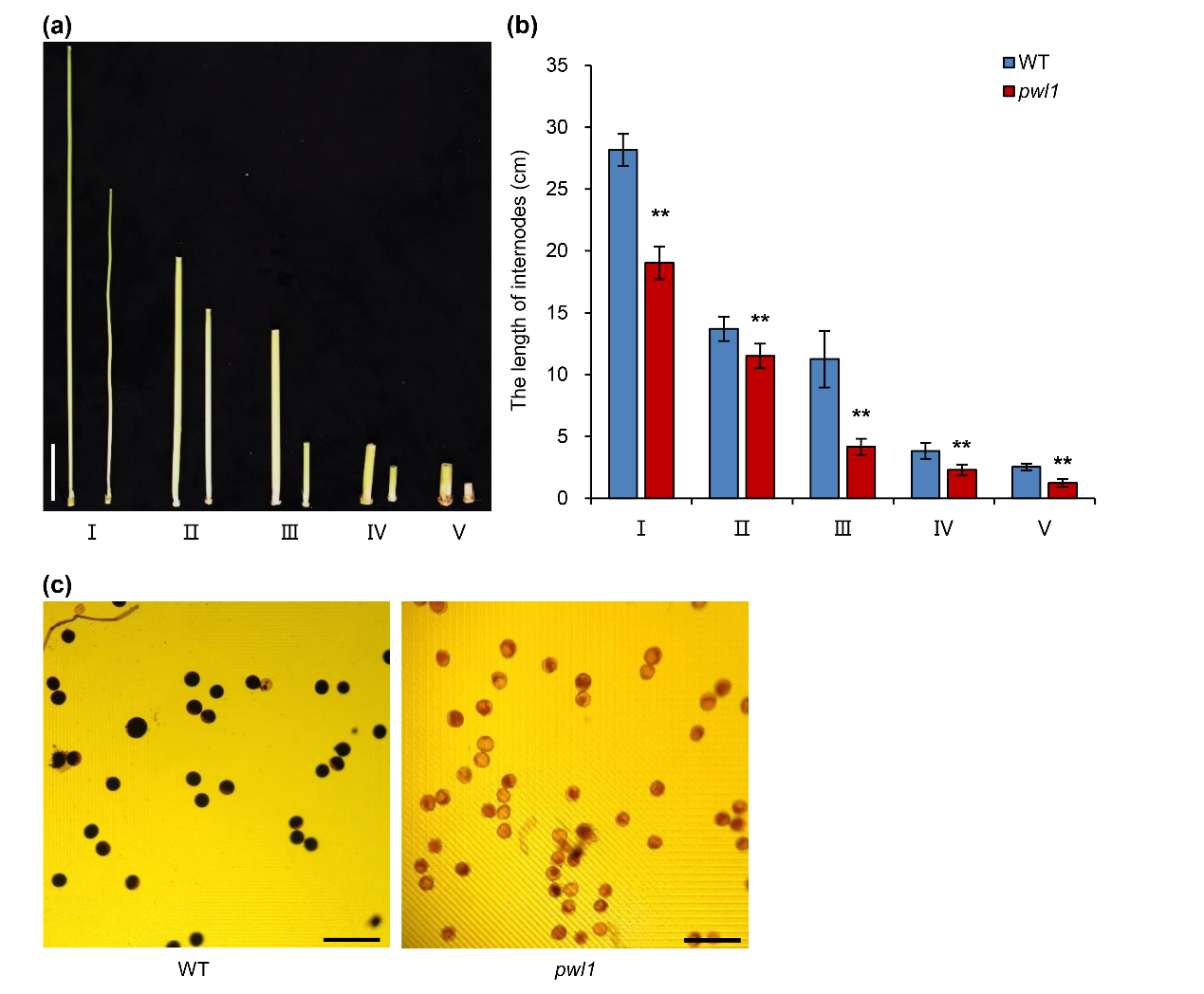


**Figure S1** Comparison of internode length and pollen grains between wild type (WT) and *pwl1*. (a) Comparison of main stems between WT (left) and *pwl1* (right). Bar, 4 cm. (b) Comparison of internode lengths of main stems between WT and *pwl1* at the mature stage. Date are means ± SD (*n* = 6). **, *P* < 0.01 (Student’s *t*-test). (c) Pollen grains of the WT and *pwl1* mutant stained by I_2_-KI solution. Bars, 200 μm.


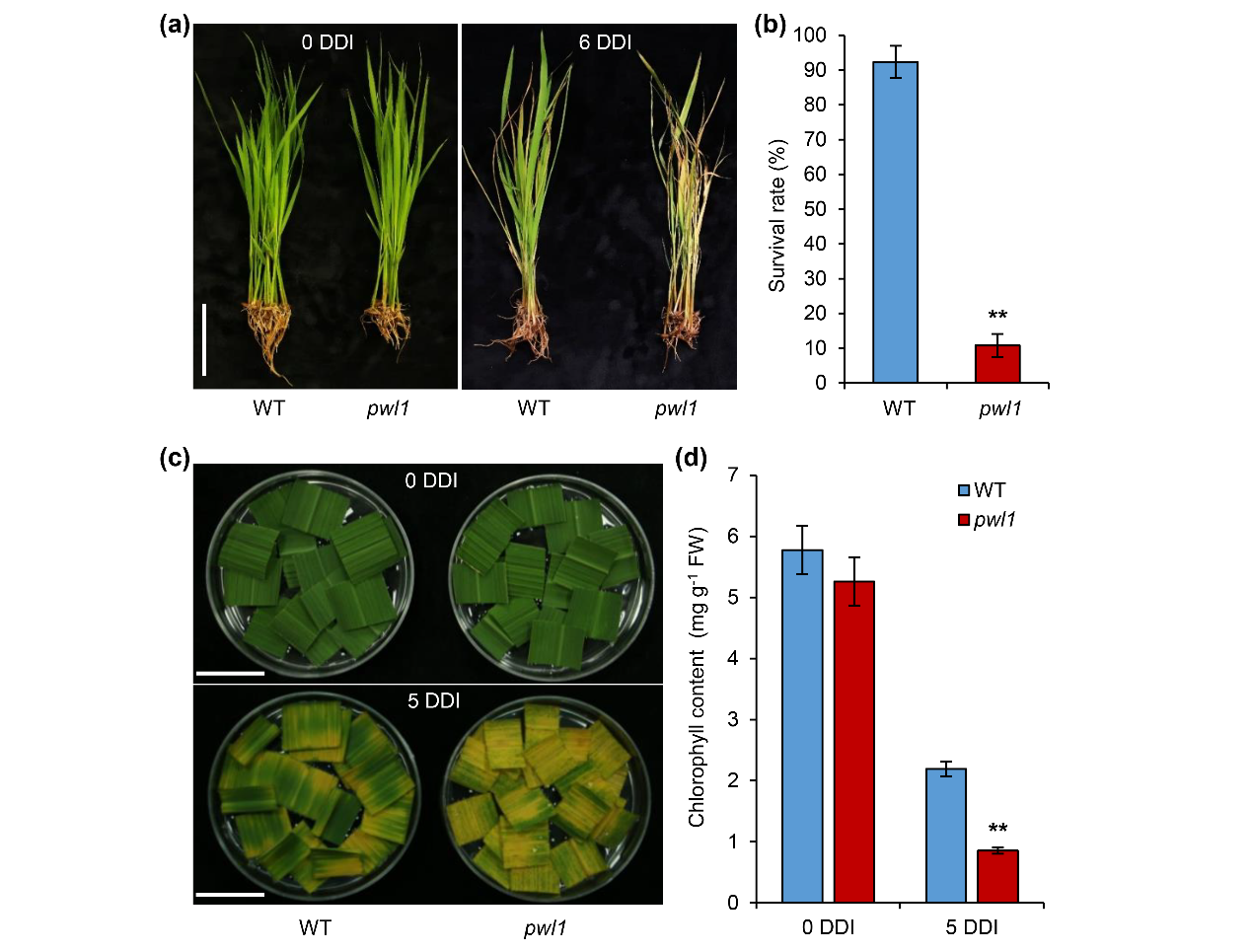


**Figure S2** Dark stress-induced senescence phenotype of *pwl1* mutant. (a) Plants at different days of dark incubation (DDI). Two-week-old seedlings of the wild-type (WT) and *pwl1* were transferred to darkness at 28°C for 6 d. Bar, 4 cm. (b) Survival rates of WT and *pwl1* seedlings after recovery from dark treatment. (c) Detached flag leaves from WT and *pwl1* at the initial tillering stage were incubated in water for 5 DDI. Bars, 3 cm. (d) Total chlorophyll contents were observed at 0 and 5 DDI. Date are means ± SD (*n* = 3). **, *P* < 0.01 (Student’s *t*-test).


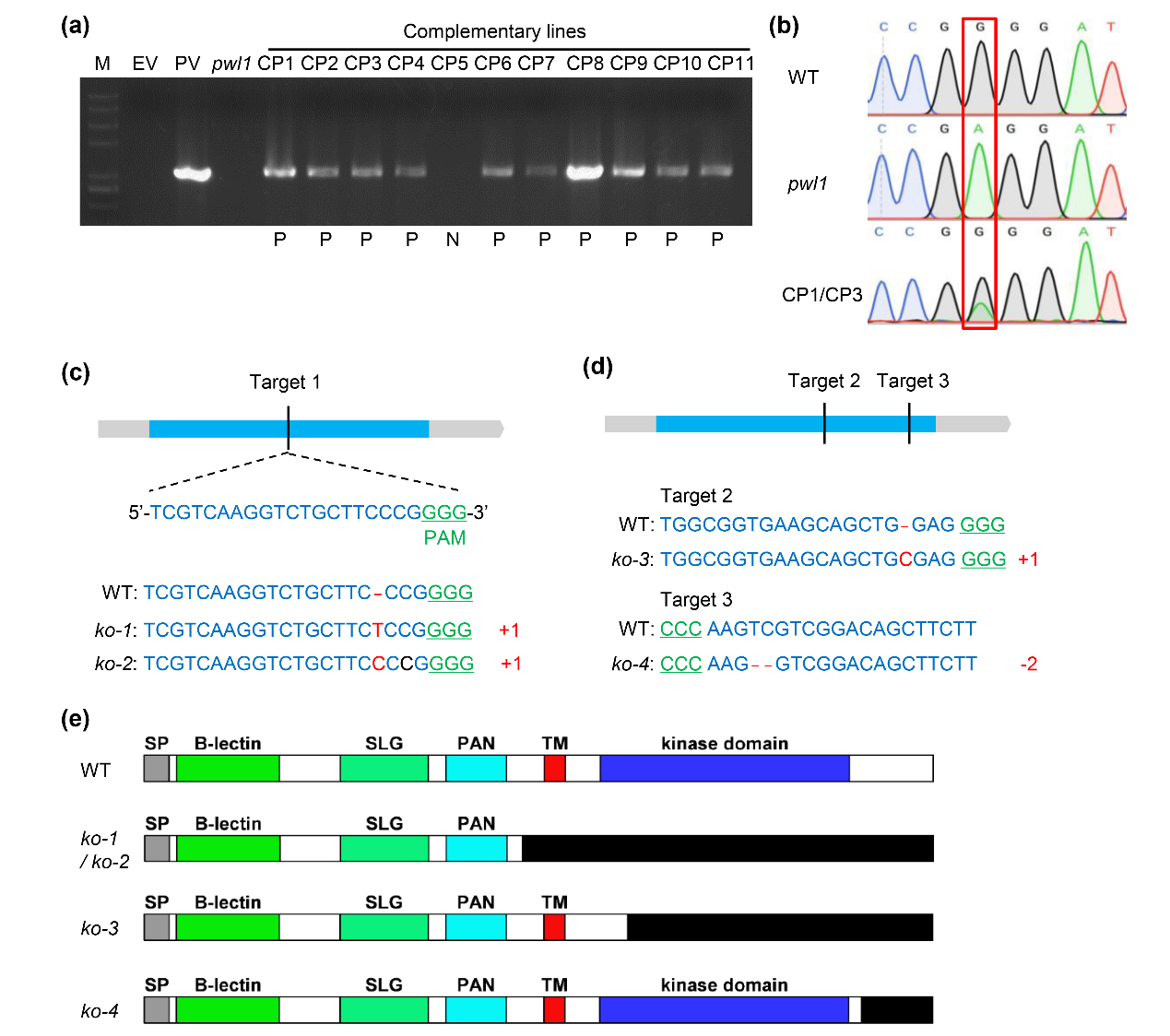


**Figure S3** Molecular identification of complementary transgenic lines and edited rice plants. (a) Genomic DNA PCR analysis of complementary transgenic lines. M: marker; EV: empty vector; PV: positive vector; P: positive plant; N: negative plant. (b) DNA sequencing analysis of the mutation site in the wild type (WT), *pwl1* and complementary transgenic lines (CP1 and CP3). (c) Mutation analysis of the CRISPR/Cas9-mediated mutants at the target site near the amino acid substitution site in *PWL1*. The sgRNA target sequence is underlined in blue, and the protospacer adjacent motif (PAM) is indicated in green. Wild type (WT) refers to the sequence of JG30. *ko-1* to *ko-2* refer to the different types of knockout mutants. Plus (+) sign indicates the number of nucleotides inserted. (d) Mutation analysis of the CRISPR/Cas9-mediated mutants at the targets in the C-terminal of *PWL1*. *ko-3* to *ko-4* refer to the different types of knockout mutants. Minus (-) sign indicates the number of nucleotides deleted. (e) Schematic diagrams of the truncated PWL1 proteins in the knockout mutants. The black boxes represent the mutated amino acids.

**
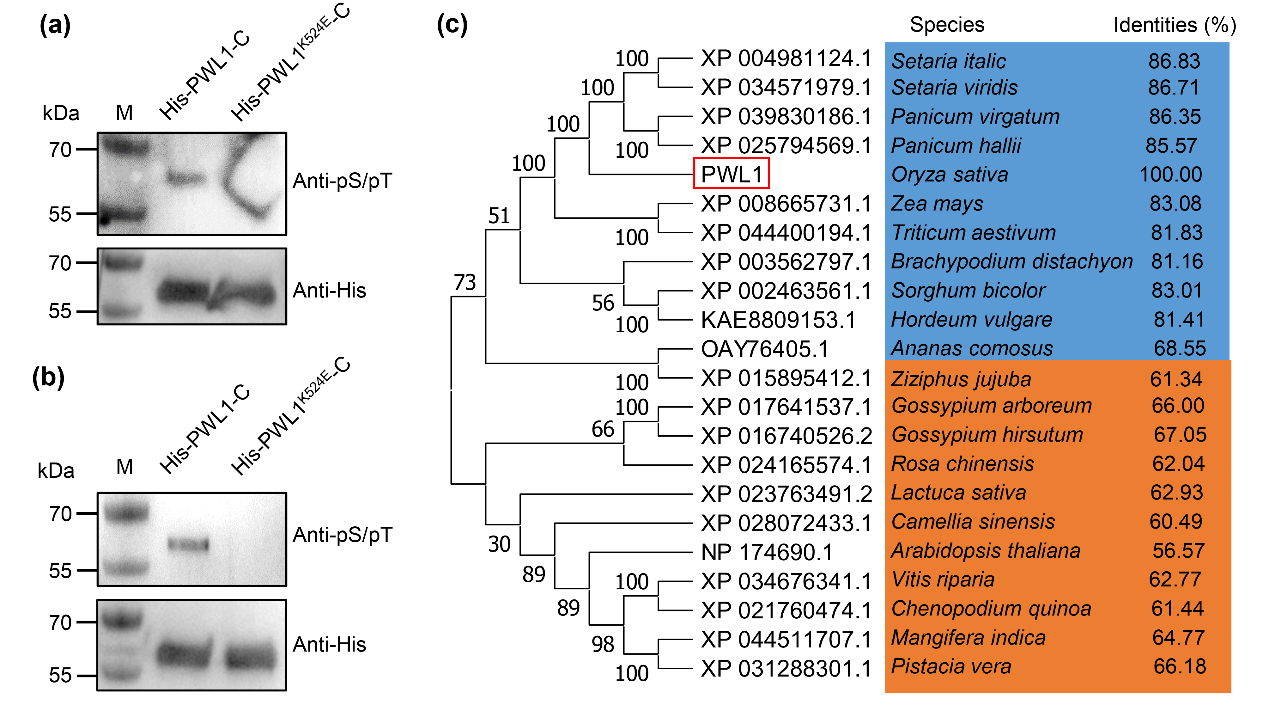
**

**Figure S4** Kinase activity and phylogenetic tree of PWL1. (a,b) The two other biological replications showing that PWL1 can be auto-phosphorylated. (c) Phylogenetic tree of the PWL1 protein. PWL1 is highlighted in red box. Monocots and dicots are in light green and blue backgrounds, respectively. The numbers on the right represent the percentage identities between PWL1 and its ortholog in corresponding species. The phylogenetic tree was constructed using MEGA 7.0 with the bootstrap method and 1,000 bootstrap replicates.


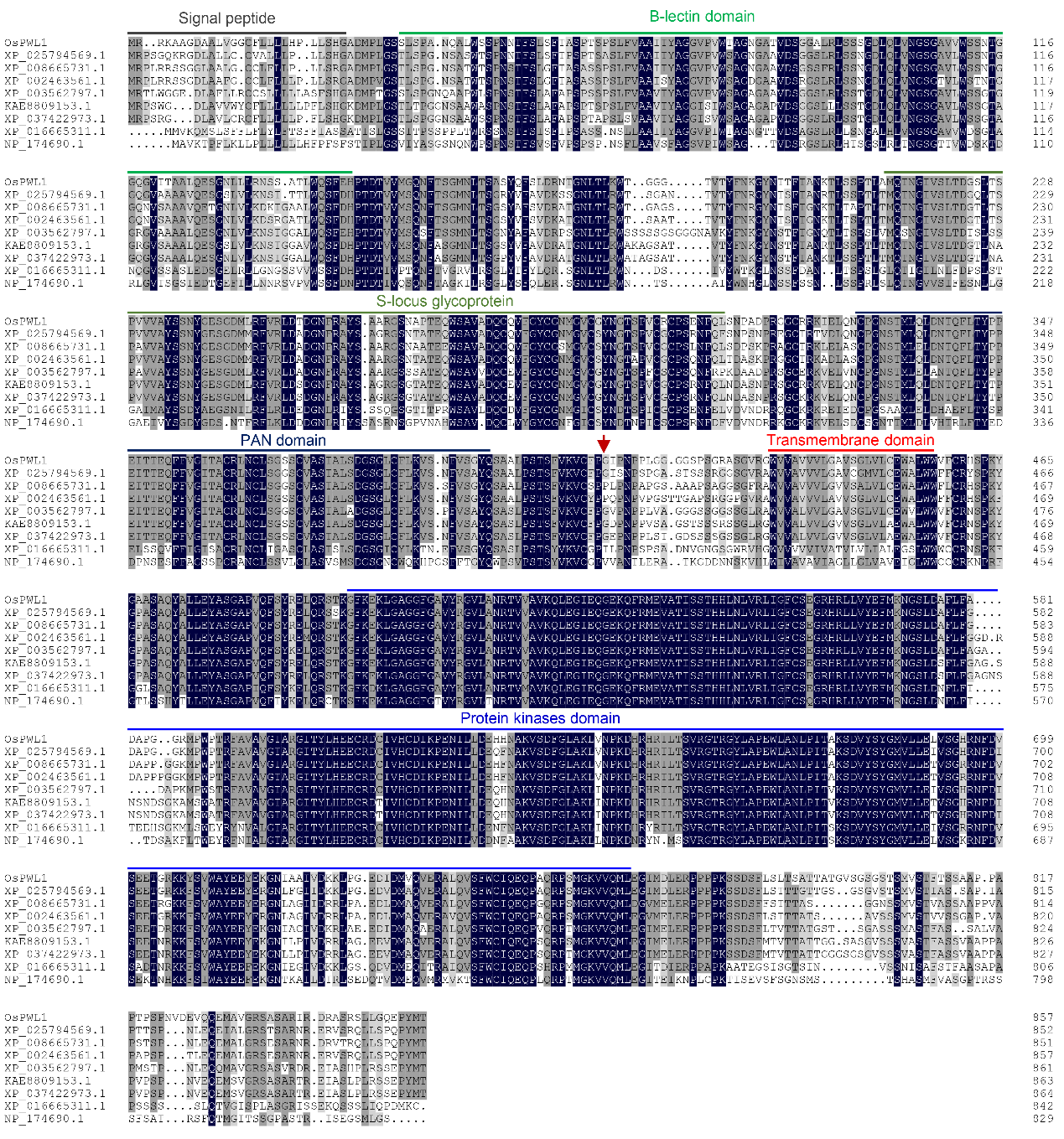


**Figure S5** Protein sequence alignment of PWL1 and its homologs from several species. Protein sequence alignment was performed among PWL1 and its homologs from *Panicum hallii* (XP_025794569.1), *Zea mays* (XP_008665731.1), *Sorghum bicolor* (XP_002463561.1), *Brachypodium distachyon* (XP_003562797.1), *Hordeum vulgare* (KAE8809153.1), *Triticum dicoccoides* (XP_037422973.1), *Gossypium hirsutum* (XP_016665311.1) and *Arabidopsis thaliana* (NP_174690.1). Arrow indicates the position of the amino acid transition.


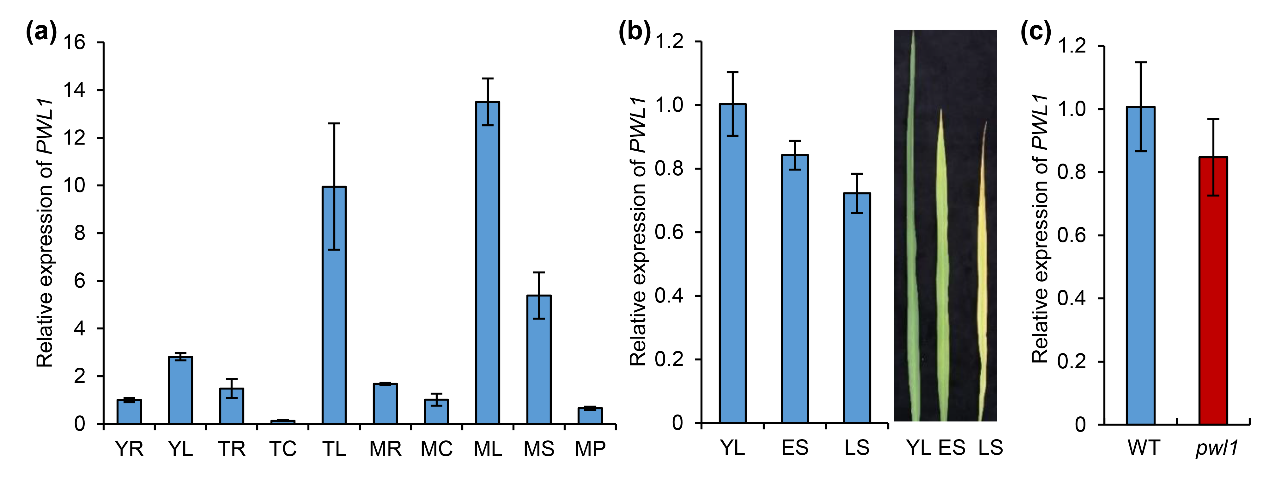


**Figure S6** Expression pattern of *PWL1*. (a) Relative expression of *PWL1* in various organs of the wild type (WT) plants at different growth stages. YR, Young root; YL, young leaf; TR, tillering root; TC, tillering culm; TL, tillering leaf; MR, mature root; MC, mature culm; ML, mature leaf; MS, mature sheath; YP, young panicle. Rice *OsActin1* was used as an internal control. Data are means ± SD (*n* = 3). (b) Relative expression of *PWL1* in various ages of WT leaves at the heading stage. Rice *OsActin1* was used as an internal control. YL, ES and LS: young leaves, early-senescing, and late-senescence, respectively. Data are means ± SD (*n* = 3). (c) The expression level of *PWL1* in WT and *pwl1* plants at the tillering stage. Rice *OsActin1* was used as an internal control. Data are means ± SD (*n* = 3).


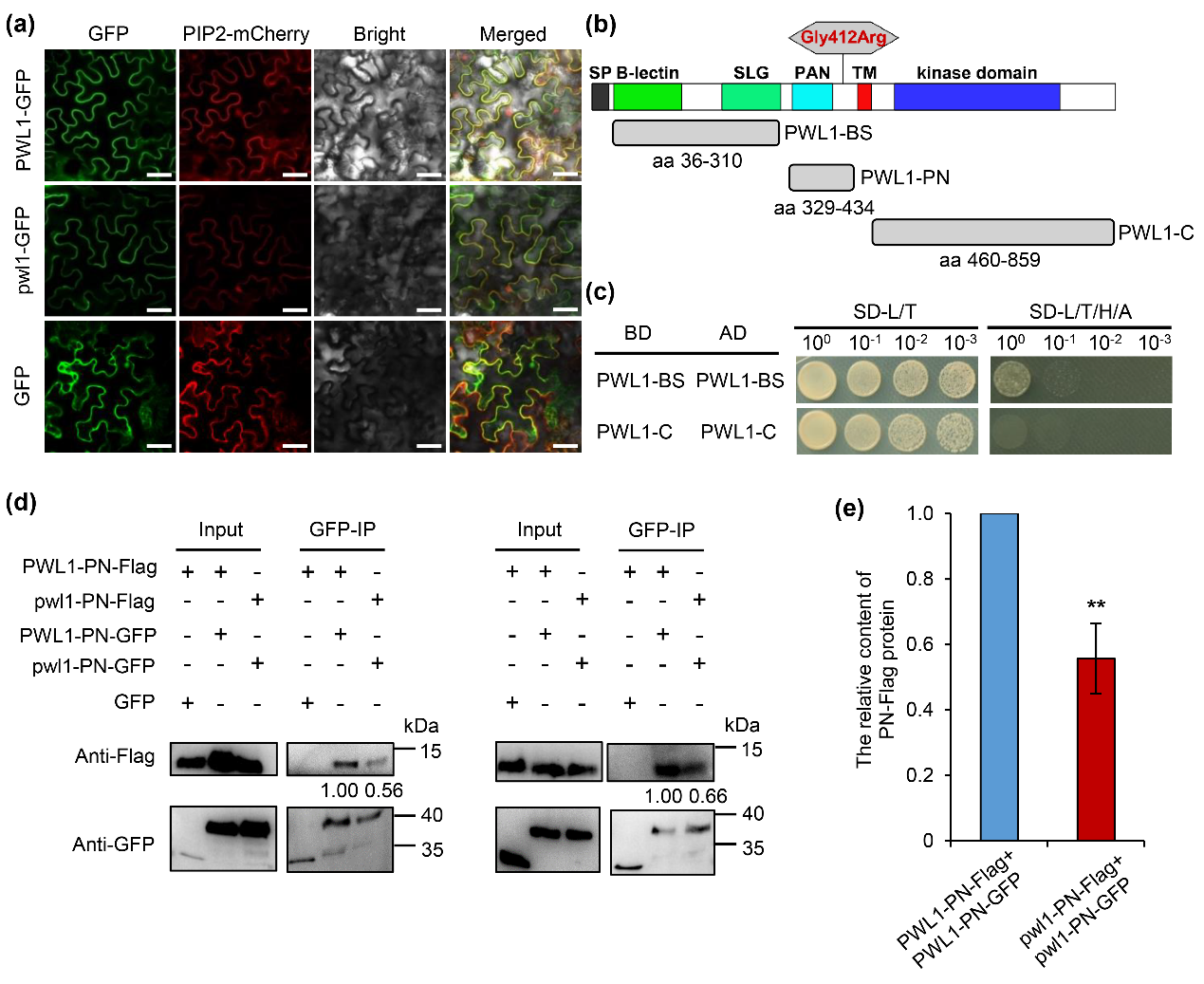


**Figure S7** The protein properties of the PWL1 protein. (a) Subcellular localization of PWL1-GFP and pwl1-GFP fusion proteins in epidermal cell of *Nicotiana benthamiana* leaves. Bars, 50 μm. (b) Sketches of various truncated of PWL1 used for yeast two-hybrid (Y2H) analysis. (c) Testing the self-interaction of two truncated PWL1 proteins using Y2H. (d) The two other biological replications of the PN fragment interacts with itself in Co-IP assay. The relative contents of proteins were measured using Image J software and indicated with numbers below the bands. (e) Quantification of the relative contents of PN-Flag proteins after IP. The relative contents of PWL1-PN-Flag proteins were defined as 1. Data are means ± SD (*n* = 3). **, *P* < 0.01 (Student’s *t*-test). Three independent biological replications were performed and used for quantification analysis.


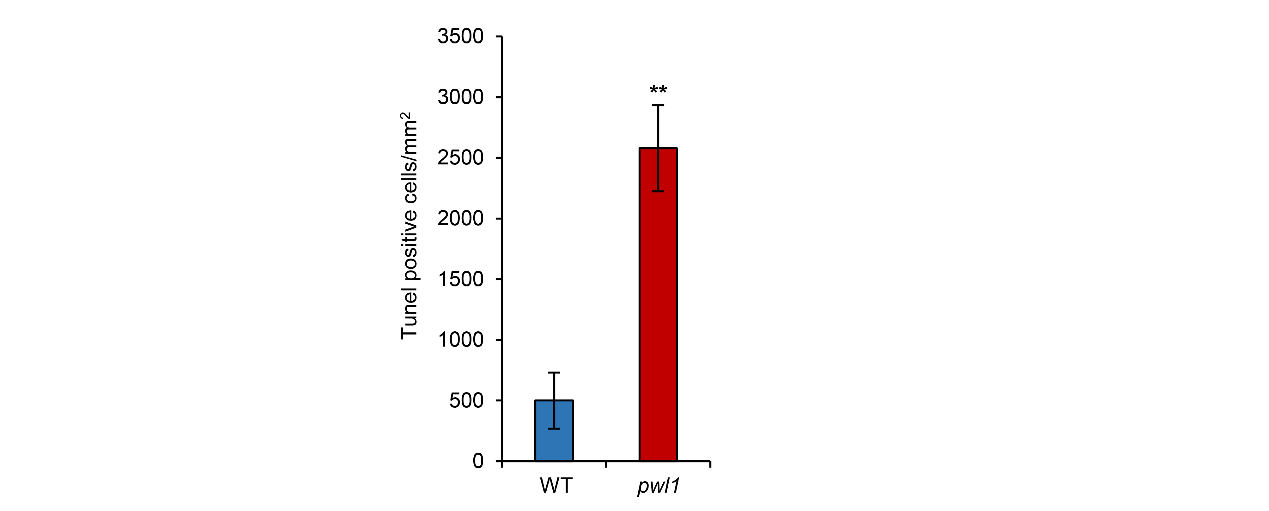


**Figure S8** Comparison of the numbers of TUNEL-positive cells in the wild type (WT) and *pwl1* mutant. Data are means ± SD (*n* = 3). **, *P* < 0.01 (Student’s *t*-test).

**
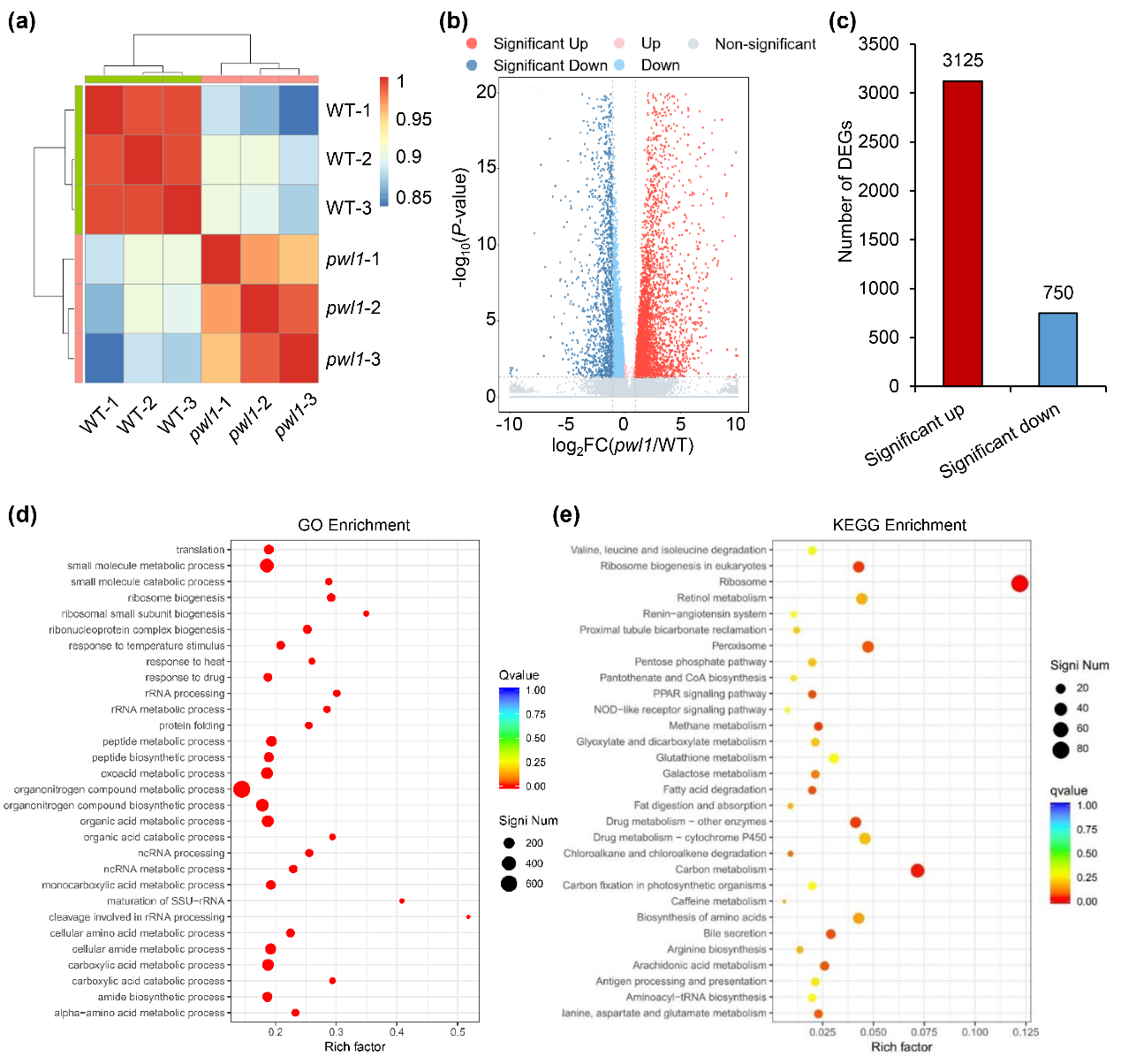
**

**Figure S9** Transcriptome analysis, Gene Ontology (GO) and Kyoto Encyclopedia of Genes and Genomes (KEGG) analysis of DEGs in wild type (WT) and *pwl1*. (a) Correlation analysis of transcriptomic data between WT and *pwl1*. Three biological replicates were used. (b) Volcano plot analysis of differentially expressed genes (DEGs) between WT and *pwl1*. Criteria for DEGs were set as *q*-value < 0.05. (c) Column chart of DEGs compared between WT and *pwl1*. (d) GO classification based on DEGs. (e) KEGG enrichment analysis of DEGs.


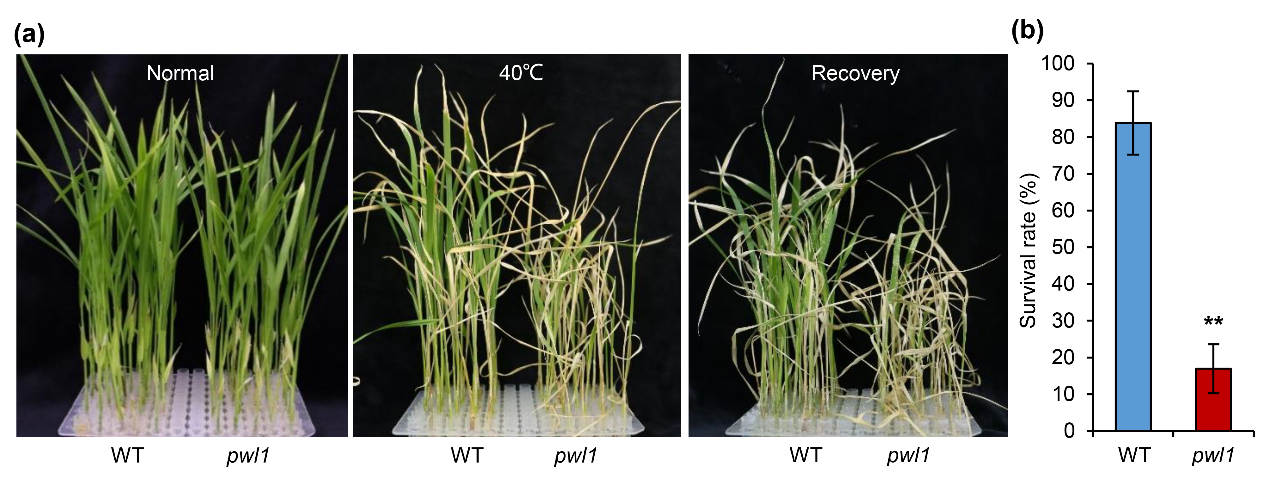


**Figure S10** The rice mutant *pwl1* was more sensitive to heat stress. (a) Phenotypes of rice seedlings before and after heat treatments. Two-week-old wild-type (WT) and *pwl1* seedlings grown at 28°C were transferred to 40°C for 7 d followed a recovery at 28°C for 7 d. (b) Survival rates of WT and *pwl1* seedlings after recovery from heat

treatment. Data are means ± SD (*n* = 3). **, *P* < 0.01 (Student’s *t*-test).

**
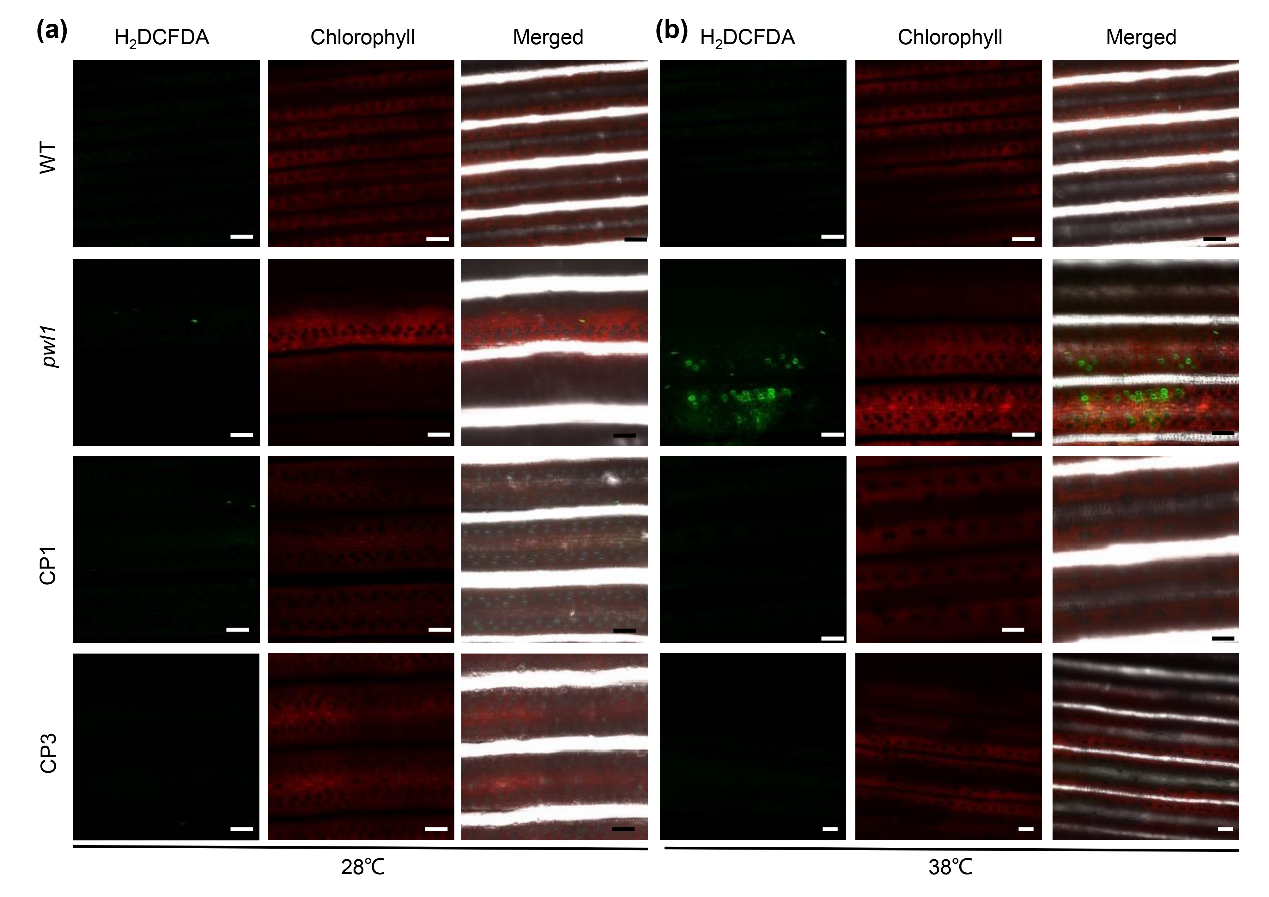
**

**Figure S11** Representative images of H_2_DCFDA fluorescence from mesophyll cells from leaves of wild type (WT) and *pwl1* measured at 28°C (a) and 38°C (b). Red, chlorophyll; green, oxidized H_2_DCFDA. Bars, 50 μm.


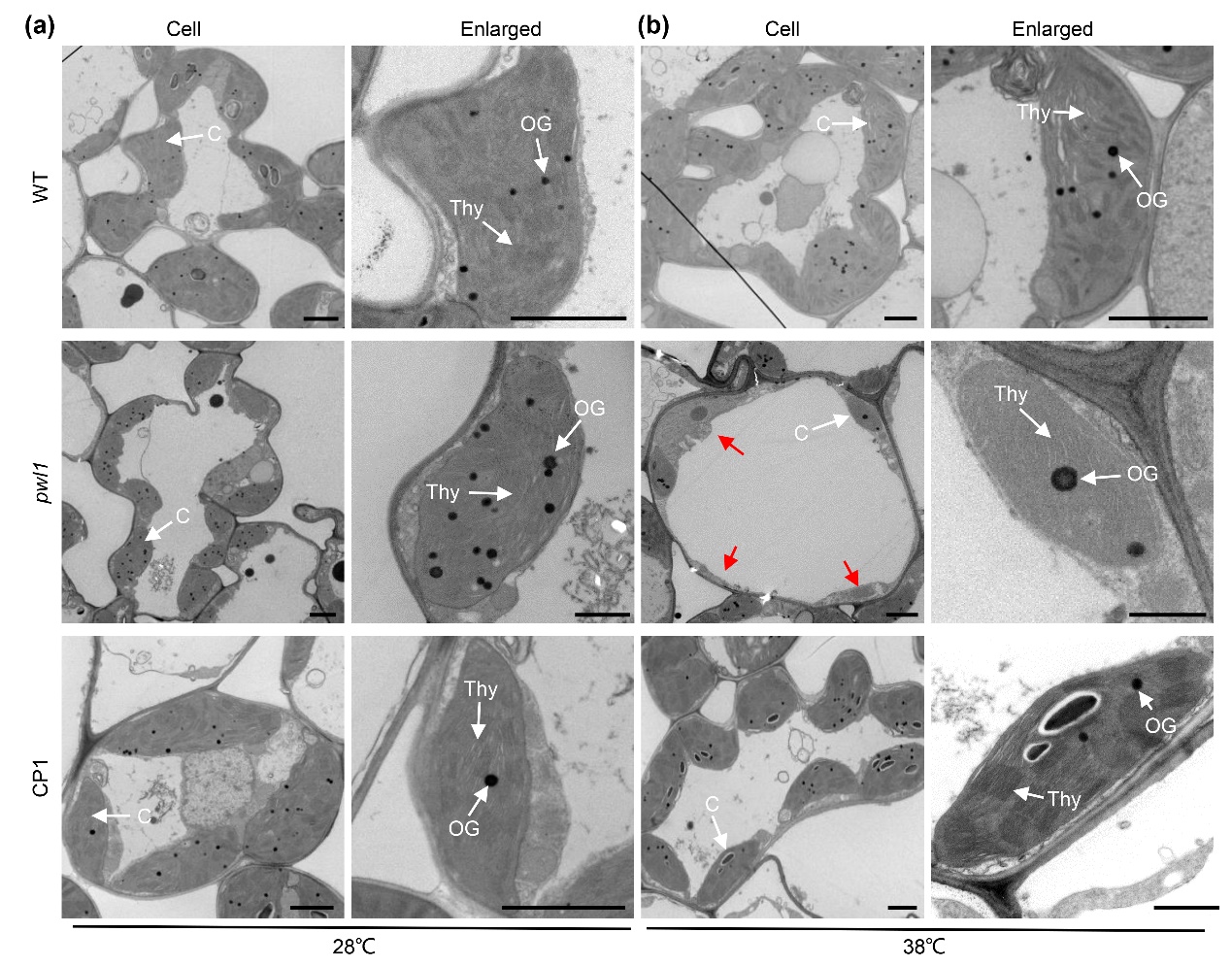


**Figure S12** Ultrastructure of chloroplasts in mesophyll cells of the wild-type (WT), *pwl1* and CP1 at 28°C (a) and 38°C (b). Bars, 2 μm.


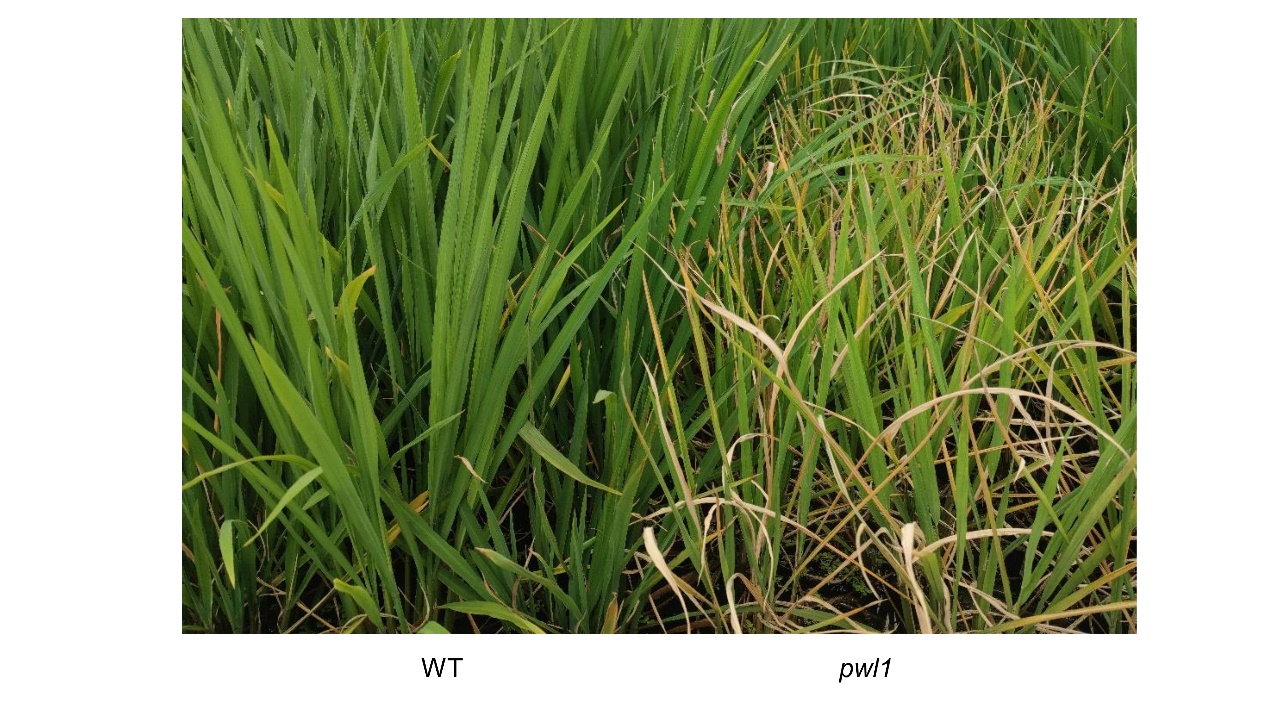


**Figure S13** Comparison of gross morphology between wild type (WT) and *pwl1* plants in the paddy field at 1-month late sowing.

**Table S1**. Comparison of major agronomic traits among the wild-type (WT), *pwl1* and the *PWL1* complemented plants (CP1 and CP3).

| Materials |  | |  | | Agronomic traits | | | | |
| --- | --- | --- | --- | --- | --- | --- | --- | --- | --- |
|  | PH (cm) | TN (No.) | | PB (No.) | | SB (No.) | PL (cm) | SR (%) | TW (g) |
| WT | 83.20±5.37 | 11.30±1.57 | | 11.10±0.74 | | 34.60±4.81 | 24.28±0.68 | 92.99±3.02 | 25.69±0.38 |
| *pwl1* | 65.27±7.25^**^ | 7.80±0.92^**^ | | 9.60±0.84^**^ | | 9.10±2.13^**^ | 19.33±0.82^**^ | 53.07±6.68^**^ | 19.23±1.00^**^ |
| CP1 | 82.40±4.58 | 11.70±1.49 | | 11.10±1.79 | | 35.00±3.92 | 24.21±0.70 | 93.40±2.27 | 25.46±0.45 |
| CP3 | 88.90±5.00 | 11.70±1.95 | | 11.30±1.83 | | 34.00±2.00 | 24.19±0.67 | 92.98±2.83 | 25.66±0.43 |

PH, Plant height; TN, tiller number; PB, number of primary branches per panicle; SB, number of secondary branches per panicle; PL, panicle length; SR, seed setting rate; TW, 1000-grain weight.

Data are means ± SD (*n* = 10). **, *P* < 0.01 (Student’s *t*-test).

**Table S2.** Genetic analysis of the *pwl1* mutant.

| Cross combination | F_1_ | |  | F_2_ | | | | |  |
| --- | --- | --- | --- | --- | --- | --- | --- | --- | --- |
|  | Wild type | *pwl1* |  | Wild type | *pwl1* | Total | *P*-value | χ^2^（3:1） |  |
| JG30/*pwl1* | 12 | 0 |  | 138 | 38 | 176 | 0.30 | 1.09 |  |
| NIP/*pwl1* | 23 | 0 |  | 261 | 75 | 336 | 0.26 | 1.29 |  |

**Table S3.** Markers used for fine mapping.

| Name | Forward sequence (5’-3’) | Forward sequence (5’-3’) |
| --- | --- | --- |
| ID3-28 | GACTCGCCCAGATTCTCTCC | GGGGTTGGGATGAAACGAAA |
| ID3-29 | GTCGTTGAGGTGAACAGGATAA | GCCTCACAACGAAAACCAGA |
| ID3-30 | AGTCCACTGTCCACTGCTAG | TGCAGCACGAACTTCAAGAC |
| ID3-34 | CTCATGGATGCAACGAGGTT | CATACTCTCCCACCCACGTT |
| ID3-36 | GGATGGGCACGAATCATTGT | GCTGGGCAATCACAATACACA |
| ID3-46 | GCTAGCAATCTCTTCCAGCAC | GATGGAGTACTTGCGTCCCT |
| ID3-50 | GGCGGCAACACGACCAGCTT | TAGTGGACCGACAATAATAT |

**Table S4.** List of open reading frames in the 85.54 kb target region.

| Gene name | Physical location | Function prediction |
| --- | --- | --- |
| LOC_Os03g62140 | 35,205,727-35,206,590 | heat shock protein DnaJ, putative, expressed |
| LOC_Os03g62150 | 35,207,543-35,208,331 | heat shock protein DnaJ, putative, expressed |
| LOC_Os03g62160 | 35,211,351-35,208,499 | expressed protein |
| LOC_Os03g62170 | 35,212,863-35,218,270 | cyclase/dehydrase family protein, expressed |
| **LOC_Os03g62180** | **35,222,920-35,218,962** | **lectin protein kinase family protein, putative, expressed** |
| LOC_Os03g62190 | 35,224,965-35,230,943 | transposon protein, putative, unclassified, expressed |
| LOC_Os03g62200 | 35,238,255-35,241,976 | ammonium transporter protein, putative, expressed |
| LOC_Os03g62210 | 35,242,074-35,242,925 | subtilisin N-terminal Region family protein, expressed |
| LOC_Os03g62220 | 35,243,970-35,243,566 | expressed protein |
| LOC_Os03g62224 | 35,248,973-35,248,256 | hypothetical protein |
| LOC_Os03g62230 | 35,251,282-35,252,606 | ZOS3-24 - C2H2 zinc finger protein, expressed |
| LOC_Os03g62240 | 35,254,202-35,253,134 | expressed protein |
| LOC_Os03g62250 | 35,264,427-35,256,091 | zinc finger, C3HC4 type domain containing protein, expressed |
| LOC_Os03g62260 | 35,264,558-35,259,267 | FAD binding domain containing protein, expressed |
| LOC_Os03g62270 | 35,266,202-35,269,749 | MATE efflux family protein, putative, expressed |
| LOC_Os03g62280 | 35,284,998-35,287,389 | expressed protein |
| LOC_Os03g62290 | 35,289,401-35,288,630 | expressed protein |

**Table S5.** Primers used for vector construction and transgenic line test.

| Name | Primer sequence (5’-3’) | Restriction enzyme |
| --- | --- | --- |
| pPWL1-CF | cgagctcggtacccggggaTTGTTTCTGCAGCACTGGTC | *Bam*HI |
| pPWL1-CR | caggtcgactctagaggatAAATAAGACCCAGTATAAGCC | *Bam*HI |
| PWL1-GFP-F | tacgaattcgagctcggtaccATGCGGCGGAAGGCGGCC | *Bam*HI |
| PWL1-GFP-R | ctcgcccttgctcacggatccCATTGTCATGTATGGCTCCTGC | *Bam*HI |
| pET28a-PWL1-CF | agcaaatgggtcgcggatccTGCCGGCACAGCCCCAAG | *Bam*HI |
| pET28a-PWL1-CR | cggagctcgaattcggatcCATTGTCATGTATGGCTC | *Bam*HI |
| PWL1- K524E-F | gAGCAGCTGGAGGGGATCGAGCAGGGGGAGAA |  |
| PWL1- K524E-R | ATCCCCTCCAGCTGCTcCACCGCCACCACCGTCCG |  |
| PWL1-gRT1 | TCGTCAAGGTCTGCTTCCCGgttttagagctagaaat |  |
| PWL1-OsU6aT1 | CGGGAAGCAGACCTTGACGACggcagccaagccagca |  |
| PWL1-KD-gRT1 | TGGCGGTGAAGCAGCTGGAGgttttagagctagaaat |  |
| PWL1-KD-OsU6aT1 | CTCCAGCTGCTTCACCGCCACggcagccaagccagca |  |
| PWL1-KD-gRT2 | AAGAAGCTGTCCGACGACTTgttttagagctagaaat |  |
| PWL1-KD-OsU6aT2 | AAGTCGTCGGACAGCTTCTTCaacacaagcggcagc |  |
| U-F | CTCCGTTTTACCTGTGGAATCG |  |
| gR-R | CGGAGGAAAATTCCATCCAC |  |
| Pps-GGL | TTCAGAGGTCTCTCTCGACTAGTATGGAATCGGCAGCAAAGG |  |
| Pgs-GGR | TTCAGAGGTCTCTAAGACTTTGGAATCGGCAGCAAAGG |  |
| PF | ACAGCTTCTTGAGCCTGACG |  |
| PR | GGTAACGCCAGGGTTTTCCCAGTCA |  |
| BD-BS-F | aggccgaattcccggggatcTCCCTCTCGCCGGCGA | *Bam*HI |
| BD-BS-R | ccgctgcaggtcgacggatccTTACAGCTGGAAGTTCTCC | *Bam*HI |
| AD-BS-F | gtaccagattacgctcatatgTCCCTCTCGCCGGCGA | *Nde*I |
| AD-BS-R | cagctcgagctcgatggatccTTACAGCTGGAAGTTCTCC | *Bam*HI |
| BD-PN-F | aggccgaattcccggggatcTGCCCGGGCAACTCCAC | *Bam*HI |
| BD-PN-R | ccgctgcaggtcgacggatccTTACCCGCGGACGCCCGACG | *Bam*HI |
| AD-PN-F | gtaccagattacgctcatatgTGCCCGGGCAACTCCAC | *Nde*I |
| AD-PN-R | cagctcgagctcgatggatccTTACCCGCGGACGCCCGACG | *Bam*HI |
| BD-PWL1-CF | aggccgaattcccggggatccagTGCCGGCACAGCCCCAAG | *Bam*HI |
| BD-PWL1-CR | ccgctgcaggtcgacggatccTTACATTGTCATGTATGGCTCCTG | *Bam*HI |
| AD-PWL1-CF | gtaccagattacgctcatatgTGCCGGCACAGCCCCAAG | *Nde*I |
| AD-PWL1-CR | cagctcgagctcgatggatccTTACATTGTCATGTATGGC | *Bam*HI |
| PWL1-NE-F | tccatcgatagtactgtcgacATGCGGCGGAAGGCGGC | *Sal*I |
| PWL1-NE-R | caacttttgctccatggtaccCATTGTCATGTATGGCTC | *Kpn*I |
| PWL1-CE-F | tccatcgatagtactgtcgacATGCGGCGGAAGGCGGC | *Sal*I |
| PWL1-CE-R | gtacatcccgggagcggtaccCATTGTCATGTATGGCTC | *Kpn*I |
| PN-GFPF | agctttcgcgagctcggtaccATGTGCCCGGGCAACTCCAC | *Kpn*I |
| PN-GFPR | ctcgcccttgctcacggatccCCCGCGGACGCCCGACGC | *Bam*HI |
| PN-Flag-F | gagctcggtacccggggatccATGTGCCCGGGCAACTCCAC | *Bam*HI |
| PN-Flag-R | atcgtccttgtaatcggatccCCCGCGGACGCCCGACGC | *Bam*HI |

**Table S6.** Primers sequences related to quantitative real-time PCR.

| Name | Forward sequence (5’-3’) | Forward sequence (5’-3’) |
| --- | --- | --- |
| OsActin | TGGCATCTCTCAGCACATTCC | TGCACAATGGATGGGTCAGA |
| PWL1 | CAAGATCGAGCTCCAGAACTG | GAAGAACTGCTCCGTCGTGATC |
| Osh36 | GCACGGAGGCGAACGA | TTGAGCGGTAGCACCCATT |
| Osl57 | ACCCTAAAGTAAATGAAGTC | CCTGCTCTTGTCTTGTTA |
| OsNAP | AACCATTTCATCGCGAACAAC | CAGTGACGATCCCTGCAAGG |
| SAG12 | CGTCAAGGACCAAGGCGATT | TTCACGGCTCCCTCCATG |
| WRKY53 | GAGCGACATCGACATCCT | TTGTGCTTGCCCTCGTAG |
| rbcS | CAGCAATGGCGGCAGGAT | AGGGCACCCACTTGGAACG |
| PsaL | GAAGGCTGTTTCACATCCATTT | ATCTCTCTCCTCTCGTCTCTTC |
| PsbP | GTTCGTGTTCTTCCGGGCTA | GCAGAATGAACTCGTAAGGTTG |
| LHCB5 | AACACCCTCAACTACTTCGG | GTTGATGATCCGGTAGTACTCG |
| PetC | TATCTGAGAAACGCTGCTGTAA | AATGCCCATATTCAGGAAGACA |
| SGR | AGGGGTGGTACAACAAGCTG | GCTCCTTGCGGAAGATGTAG |
| RCCR1 | CGCATTTCCTCATGGAATTT | CTTCTCACGCTGTTTGTCCA |
| RLS1 | TGTCCGACCTTGGGCTGTT | CCTTCTTCCCTTTGCCTTTCT |
| OsPAO | AAGCCTCCGATGTTACCGAA | CGAGGGTTTCCAGAATTTGA |
| HEMA | CGCTATTTCTGATGCTATGGGT | TCTTGGGTGATGATTGTTTGG |
| OsPORA | ATGGCTCTCCAAGTTCAG | TGGCTCACGCTAAGGAAC |
| OsPORB | CCGCAAGGAGGGAGCGGTG | CCCTCTTGGTGCTAAGGCCG |
| OsCGA1 | CACAGAACCCGATATCCAAGG | GCCCTCATCAAATTAACGGTAC |
| OsCHLH | AACTGGATGAGCCAGAAGAGA | AAATGCAAAAGACTTGCGACT |
| Cab1R | AGATGGGTTTAGTGCGACGAG | TTTGGGATCGAGGGAGTATTT |

**Table S7.** Differentially expressed of SAGs, photosynthesis, chloroplast metabolism and ROS generation-related genes in *pwl1* and wild-type (WT) plants.

| Category | Locus | Annotation | Log_2_ ratio |
| --- | --- | --- | --- |
| SAGs | LOC_Os05g39770 | Aminotransferase, Osh36 | 2.261 |
|  | LOC_Os02g57260 | 3-ketoacyl-CoA thiolase, OsI57 | 2.099 |
|  | LOC_Os03g21060 | NAC domain-containing protein, OsNAP | 3.708 |
|  | LOC_Os06g46270 | NAC domain-containing protein, ONAC011 | 3.726 |
|  | LOC_Os04g13140 | Senescence-specific cysteine protease, OsSAG12 | 1.768 |
|  | LOC_Os05g04640 | WRKY transcription factor, WRKY5 | 2.168 |
|  | LOC_Os05g27730 | WRKY transcription factor, WRKY53 | 1.858 |
|  | LOC_Os05g27730 | WRKY transcription factor, WRKY72 | 3.641 |
| Photosynthesis | LOC_Os12g17600 | Ribulose bisphosphate carboxylase small chain, rbcS | -1.734 |
|  | LOC_Os12g23200 | Photosystem I PsaL | -1.246 |
|  | LOC_Os09g30340 | Photosystem I PsaG/PsaK | -1.607 |
|  | LOC_Os01g59090 | Photosystem II PsbP | -1.358 |
|  | LOC_Os01g64960 | Photosystem II subunit PsbS | -1.045 |
|  | LOC_Os03g21560 | Photosystem II Pbs27 | -1.119 |
|  | LOC_Os11g13890 | Light-harvesting complex II protein, LHCB5 | -1.233 |
|  | LOC_Os07g37030 | Cytochrome b6-f complex iron-sulfur subunit, PetC | -1.212 |
| Chlorophyll degradation | LOC_Os09g36200 | senescence-inducible chloroplast stay-green protein, SGR | 1.713 |
|  | LOC_Os10g25030 | Red chlorophyll catabolite reductase, RCCR1 | 2.902 |
|  | LOC_Os02g10900 | NB-ARC domain containing protein, RLS1 | 1.374 |
|  | LOC_Os06g24730 | Hydrolase, NYC3 | 0.231 |
|  | LOC_Os03g05310 | Pheophorbide a oxygenase, PAO | 0.300 |
| Chlorophyll  synthesis | LOC_Os01g17170 | Magnesium-protoporphyrin IX monomethyl ester cyclase, YGL8 | -1.128 |
|  | LOC_Os10g35840 | Shikimate/quinate 5-dehydrogenase, HEMA | -1.550 |
|  | LOC_Os04g58200 | Protochlorophyllide reductase A, PORA | -1.026 |
|  | LOC_Os10g35370 | Oxidoreductase, PORB | -0.282 |
|  | LOC_Os06g40080 | Heme oxygenase 1, YGL2 | -0.743 |
| Chloroplast development | LOC_Os02g12790 | GATA zinc finger domain containing protein, OsCGA1 | -1.996 |
|  | LOC_Os03g20700 | Magnesium-chelatase, OsCHLH | -0.617 |
|  | LOC_Os03g48040 | 2Fe-2S iron-sulfur cluster binding domain containing protein, OsFdC2 | -0.304 |
|  | LOC_Os05g51450 | Putative Clp protease homologue, CLP8 | -0.685 |
|  | LOC_Os09g17740 | Chlorophyll A-B binding protein, Cab1R | -0.585 |
| ROS generation | LOC_Os08g35210 | Ferric reductase, OsrbohE | 3.528 |
|  | LOC_Os09g26660 | Ferric reductase, OsrbohB | 4.103 |
|  | LOC_Os11g33120 | Ferric reductase, Osrboh8 | 1.658 |
