# Supplementary figures and images for: PWL1, a G‐type lectin receptor‐like kinase, positively regulates leaf senescence and heat tolerance but negatively regulates resistance to Xanthomonas oryzae in rice
Source: Plant Biotechnol J. 2023 Aug 14;21(12):2525–45. doi: 10.1111/pbi.14150 (PMC10651159; doi:10.1111/pbi.14150)

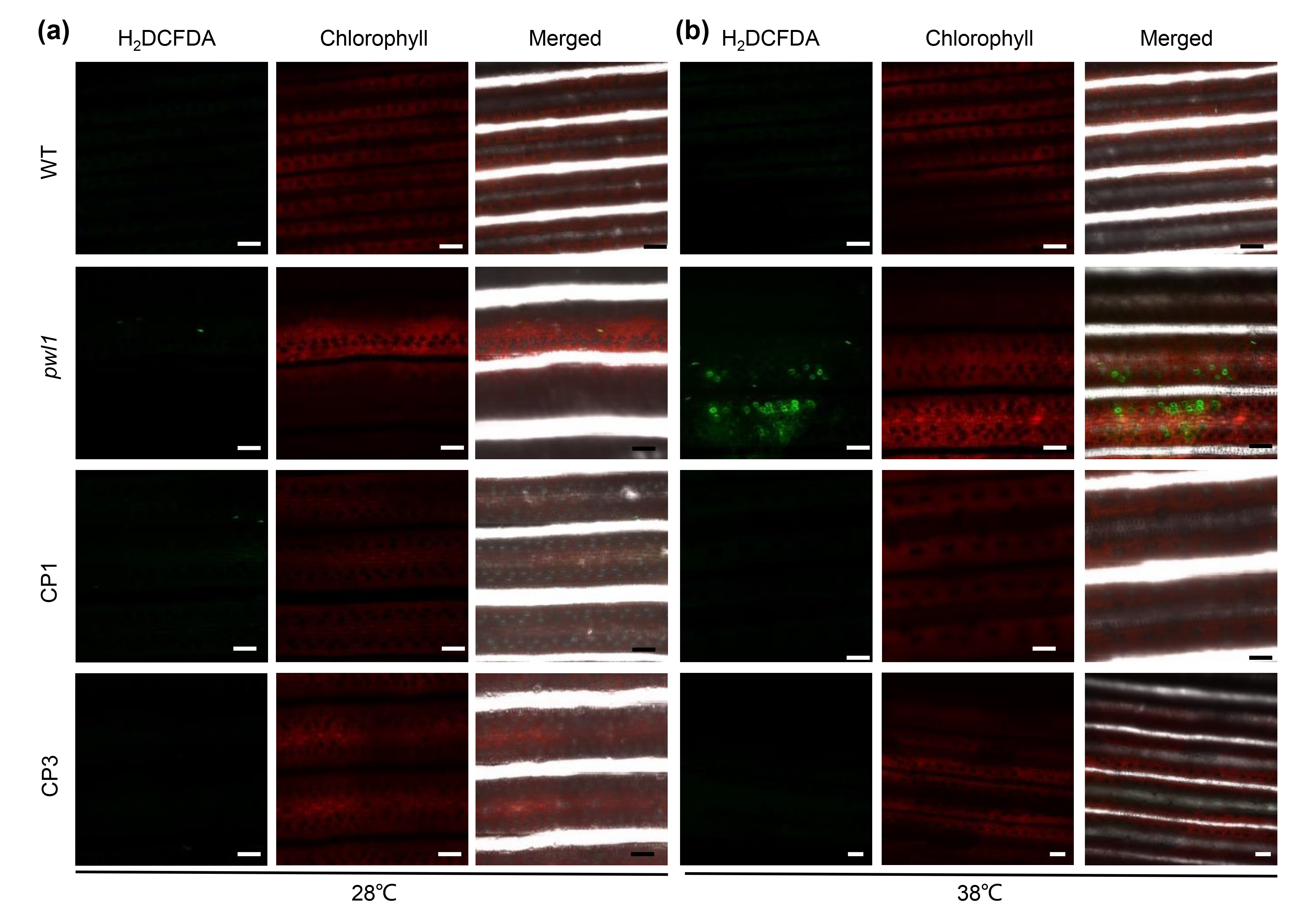

Supplement: Supplementary file 1 — Figure S1 Comparison of internode length and pollen grains between wild type (WT) and pwl1. Figure S2 Dark stress‐induced senescence phenotype of pwl1 mutant. Figure S3 Molecular identification of complementary transgenic lines and edited rice plants. Figure S4 Kinase activity and phylogenetic tree of PWL1. Figure S5 Protein sequence alignment of PWL1 and its homologues from several species. Figure S6 Expression pattern of PWL1. Figure S7 The protein properties of the PWL1 protein. Figure S8 Comparison of the numbers of TUNEL‐positive cells in the wild type (WT) and pwl1 mutant. Figure S9 Transcriptome analysis, gene ontology (GO) and Kyoto Encyclopedia of Genes and Genomes (KEGG) analysis of DEGs in wild type (WT) and pwl1. Figure S10 The rice mutant pwl1 was more sensitive to heat stress. Figure S11 Representative images of H2DCFDA fluorescence from mesophyll cells from leaves of wild‐type (WT) and pwl1 plants measured at 28 °C (a) and 38 °C (b). Figure S12 Ultrastructure of chloroplasts in mesophyll cells of the wild type (WT), pwl1 and CP1 at 28 °C (a) and 38 °C (b). Figure S13 Comparison of gross morphology between wild‐type (WT) and pwl1 plants in the paddy field at 1‐month late sowing. Table S1 Comparison of major agronomic traits among the wild‐type (WT), pwl1 and the PWL1 complemented plants (CP1 and CP3). Table S2 Genetic analysis of the pwl1 mutant. Table S3 Markers used for fine mapping. Table S4. List of open reading frames in the 85.54‐kb target region. Table S5 Primers used for vector construction and transgenic line test. Table S6 Primers sequences related to quantitative real‐time PCR. Table S7 Differentially expressed of SAGs, photosynthesis, chloroplast metabolism and ROS generation‐related genes in pwl1 and wild‐type (WT) plants. [file PBI-21-2525-s001.zip › Figure S11.tif]
